# euler*APE*: Drawing Area-Proportional 3-Venn Diagrams Using Ellipses

**DOI:** 10.1371/journal.pone.0101717

**Published:** 2014-07-17

**Authors:** Luana Micallef, Peter Rodgers

**Affiliations:** School of Computing, University of Kent, Canterbury, Kent, United Kingdom; University of Ulm, Germany

## Abstract

Venn diagrams with three curves are used extensively in various medical and scientific disciplines to visualize relationships between data sets and facilitate data analysis. The area of the regions formed by the overlapping curves is often directly proportional to the cardinality of the depicted set relation or any other related quantitative data. Drawing these diagrams manually is difficult and current automatic drawing methods do not always produce appropriate diagrams. Most methods depict the data sets as circles, as they perceptually pop out as complete distinct objects due to their smoothness and regularity. However, circles cannot draw accurate diagrams for most 3-set data and so the generated diagrams often have misleading region areas. Other methods use polygons to draw accurate diagrams. However, polygons are non-smooth and non-symmetric, so the curves are not easily distinguishable and the diagrams are difficult to comprehend. Ellipses are more flexible than circles and are similarly smooth, but none of the current automatic drawing methods use ellipses. We present euler*APE* as the first method and software that uses ellipses for automatically drawing accurate area-proportional Venn diagrams for 3-set data. We describe the drawing method adopted by euler*APE* and we discuss our evaluation of the effectiveness of euler*APE* and ellipses for drawing random 3-set data. We compare euler*APE* and various other methods that are currently available and we discuss differences between their generated diagrams in terms of accuracy and ease of understanding for real world data.

## Introduction

Data is routinely generated and analysed. For instance, relationships between groups of genes are studied to understand biological processes, improve health care, find cures to illnesses, and solve problems in agriculture. To aid analysis, Venn diagrams are often used. Each data set is represented by a closed curve and each set relation is represented by one of the spatial relationships between the curves. Both the curves and their spatial relationships are often easily visible, as closed curves are processed preattentively and pop out as complete distinct objects [1], particularly when the curves are smooth and have good continuation [2]. Closed curves also aid set analysis due to the perceptual grouping principles of common regions [3] and closure [4].

A Venn diagram with *n* curves is referred to as an *n*-Venn diagram and its regions depict all of the 2*^n^* different combinations of the curve overlaps. A Venn diagram can also be *area-proportional*, such that the area of each region in the diagram is directly proportional to quantitative data corresponding to the depicted set relation [5]. Size is processed preattentively [6] and is easily noticeable due to its pop-out effect [7]. Thus, a Venn diagram can easily depict the data set relations as well as their cardinality or other associated quantitative data. Small multiples of such diagrams can also facilitate the analysis of a collection of data sets for different attributes (e.g., Venn diagrams depicting overlapping disease symptoms for different countries [8]; [9], [10]).

**Figure 1:**
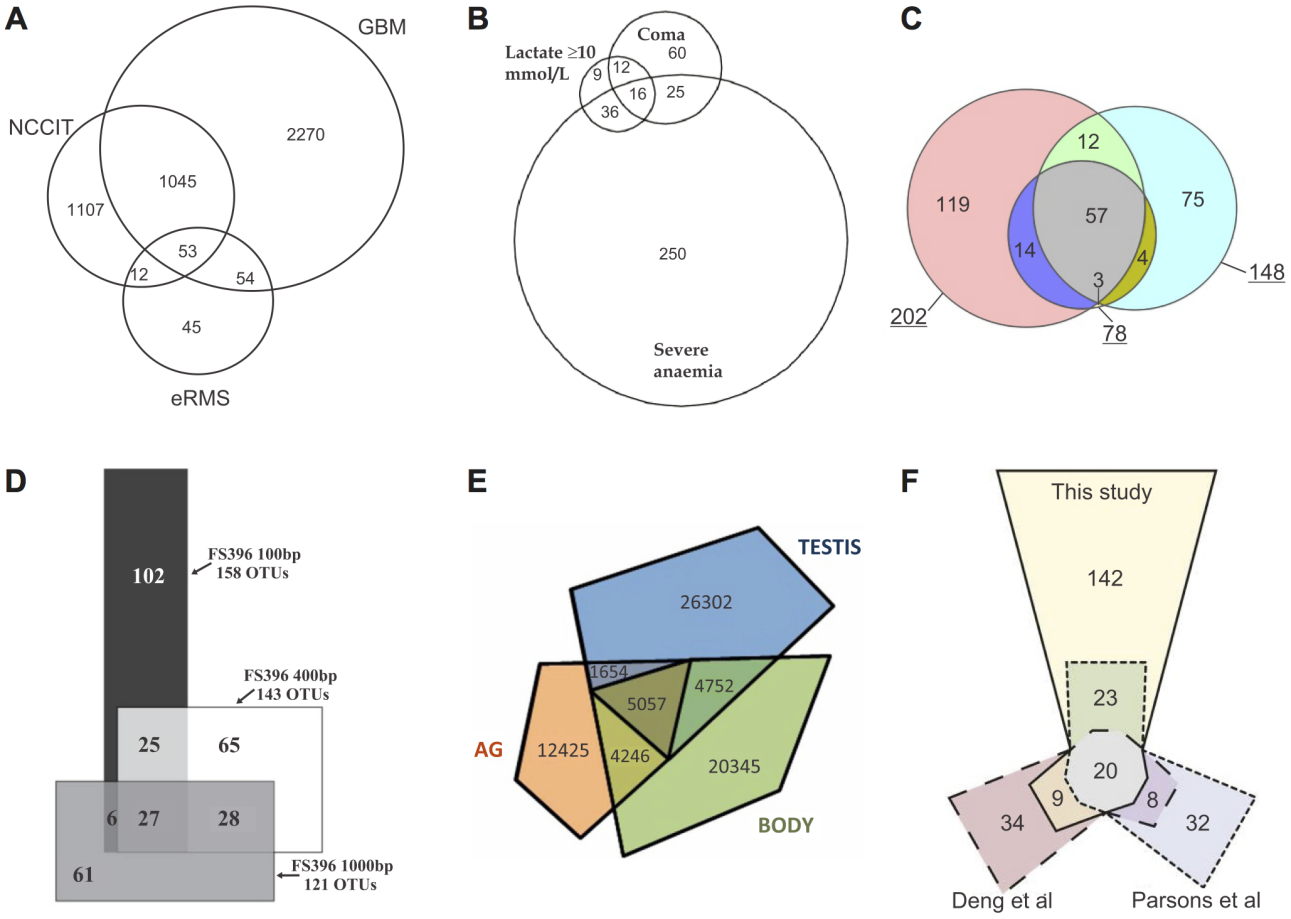
Examples of area-proportional 3-Venn diagrams drawn with circles (A–C) and polygons (D–F) in literature. (A) Comparing the cell-type of differentially regulated genes after an anti-cancer drug treatment [11]. The method used to draw the diagram has not been noted. This diagram is a reprint of Figure 3B in [11], previously published under a CC BY license. (B) Summarizing prognostic indicators of severe malaria [12]. The method used to draw the diagram has not been noted. This diagram is a reprint of Figure 3 in [12] (with the *N* value and the percentages in parenthesis removed), previously published under a CC BY license. (C) Analysing differences and similarities between identified chicken egg white proteome in three different studies [13]. Drawn using Venn Diagram Plotter [http://omics.pnl.gov/software/VennDiagramPlotter.php]. This diagram is a reprint of Figure 2A in [13], previously published under a CC BY license. (D) Analysing overlaps between gene libraries [14]. Drawn using DrawVenn [5]. This diagram is a reprint of Figure 4B in [14] under a CC BY license, with permission from John Wiley and Sons, original copyright 2009. (E) Studying transcriptome variation of different tissue types of the male field cricket, namely testis, accessory gland and the rest of the body [15]. Drawn using Convex Venn-3 [51]. This diagram is a reprint of Figure 1 (left) in [15] under a CC BY license, with permission from Nathan Bailey, original copyright 2013. (F) Summarizing genes affecting Top1-induced DNA damage identified in three different studies [16]. Drawn using DrawEuler [55]. This diagram is a reprint of Figure 3A in [16] (with added numeric labels indicating the quantitative data that according to the article each region in the diagram should represent), previously published under a CC BY license.

Consequently, area-proportional 3-Venn diagrams have been used to, for instance: compare the cell-type of differentially regulated genes after an anti-cancer drug treatment [11] (Figure 1A); summarize prognostic indicators of severe malaria [12] (Figure 1B); analyse differences and similarities between chicken egg white proteome in three different studies [13] (Figure 1C) and between gene libraries [14] (Figure 1D); study transcriptome variation of different tissue types of the male field cricket [15] (Figure 1E); summarize genes affecting DNA damage in three different studies [16] (Figure 1F). Such diagrams have also been used in various other disciplines, such as: neuroscience [17]; biosciences [18]; microbiology [19]; botany [20]; ecology [21]; public health [22]; museum conservation [23]; criminology [24]; information search and filtering [25]. Scientific work that specifically focuses on the generation of an area-proportional Venn diagram for the quantification of the relationships between studied data sets is also available (e.g., [26]–[28]).

An informal study identified various area-proportional Venn diagrams in the world's most cited journals (e.g., Nature) [29]. Almost all of these diagrams have two or three curves and are drawn using circles. Most of those with three circles are misleading and depict the required data inappropriately, like Figure 1A–C. For instance: in Figure 1A, the region with value 45 is bigger than those with value 53 and 54; in Figure 1B, the region with value 25 is bigger than that with value 36; in Figure 1C, the region with value 3 is much smaller than that with value 4. In some cases, the generated diagrams do not depict all the required overlaps between the curves, as demonstrated in Section 4.3 and Section 4.4.

Such area-proportional Venn diagrams cannot be drawn analytically using a specific curve shape and so numerical methods or heuristics are required [30]. Circles can draw Venn diagrams with region areas that are proportional to any data with two sets [5], but not three [30] due to their limited degrees of freedom (i.e., a centre and a radius). Polygons can draw accurate area-proportional Venn diagrams for any data with three sets [30], but as shown in Figure 1D–F, their non-smooth and non-symmetric curves are not easily distinguishable and impede comprehension [31], [32]. Despite these problems, current drawing methods use either circles or polygons.

**Figure 2:**
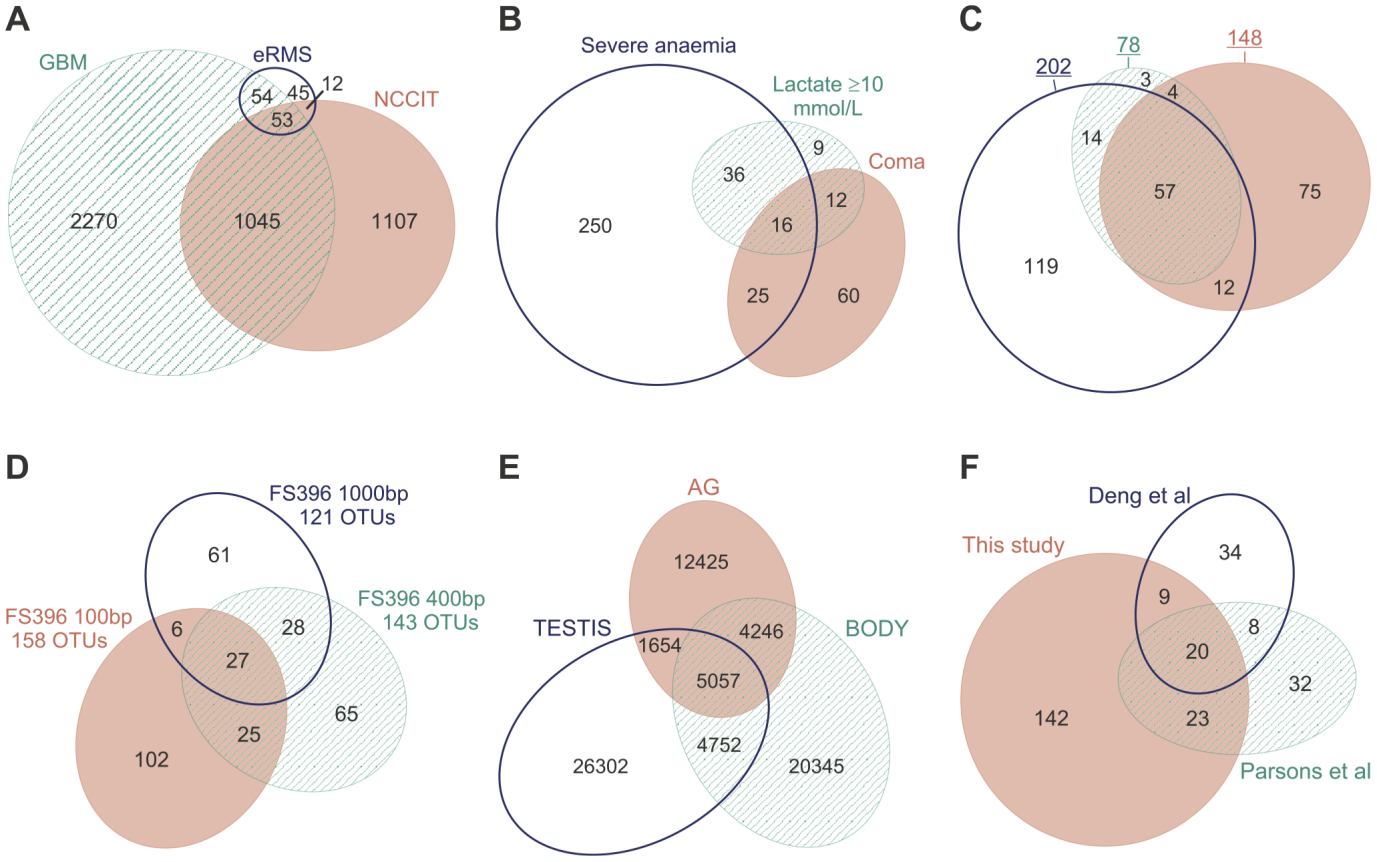
Accurate area-proportional 3-Venn diagrams drawn with ellipses and euler*APE* for the data in Figure 1. Each of these diagrams depicts the sets and the quantitative data indicated by the numeric labels in the regions of the corresponding diagram in Figure 1. These diagrams have been drawn with euler*APE*, but the labels have been added manually.

Ellipses have more degrees of freedom (i.e., a centre, two semi-axes, an angle of rotation) than circles and are similarly smooth. So diagrams drawn with ellipses are more likely to be accurate with respective to the required quantitative data and easy to comprehend due to their distinguishable curves. This is illustrated in Figure 2 where the diagrams accurately depict the quantities indicated by the numeric labels of the respective diagram in Figure 1. The diagrams in Figure 2 were drawn using our novel drawing method and software, euler*APE*, which is the first to use ellipses.

The benefits of ellipses was noted earlier (in 2004 in the first paper on area-proportional Venn diagrams [5] and later [29], [30]), but ellipses were never adopted due to difficulties in calculating the area of the regions of overlapping ellipses and in adjusting the various properties of the ellipses [5]. Thus, euler*APE* is the first to address this well-known, difficult problem. It is also novel, in that it is neither an extension nor an adaptation of any other previous drawing method. The current applications of euler*APE* are extensive and diverse. Diagrams generated by euler*APE* have appeared in numerous journal articles from diverse disciplines (e.g., [33]–[35]). A New York Times Science article (http://www.nytimes.com/interactive/2013/04/16/science/disease-overlap-in-elderly.html) cites euler*APE*, and the Pacific Northwest National Laboratory recommends it on their Venn diagram drawing software webpage (http://omics.pnl.gov/software/VennDiagramPlotter.php). This work is also the first to assess the effectiveness of ellipses in drawing accurate diagrams for the required set relations and associated quantitative data. We discuss the availability of euler*APE* in Section 3.5.

Our contributions include: (1) an optimization method to automatically draw accurate diagrams with ellipses comprised of (1a) a novel cost function to direct the optimization process (Section 3.2), (1b) a method to generate a rational starting (Section 3.3), and (1c) a mechanism to adjust the properties of the ellipses in search for a good solution (Section 3.4); (2) evaluation of (2a) the effectiveness of euler*APE* in drawing an accurate diagram when one is known to exist for the given 3-set data (Section 4.1), (2b) the effectiveness of euler*APE* and ellipses in drawing an accurate diagram for random 3-set data for which an accurate diagram drawn with ellipses might not exist and the comparison of these diagrams with those generated by a variant of euler*APE* that restricts the ellipses to circles (Section 4.2), (2c) the effectiveness of euler*APE* and venneuler [29] (the latest proposed circle-based method) in generating accurate Venn diagrams for 3-set data (Section 4.3), and (2d) the quality of the diagrams generated by euler*APE* and various other drawing methods that use circles or polygons in depicting real world medical data (Section 4.4).

All the experiments mentioned in this article were run on an Intel Core i7-3770 CPU @3.4GHz with 8GB RAM, 64-bit Microsoft Windows 7 Professional SP1 and Java Platform 1.7.0_10.

## Current Automatic Drawing Methods and Software

### 2.1. Circle-based

**Figure 3:**
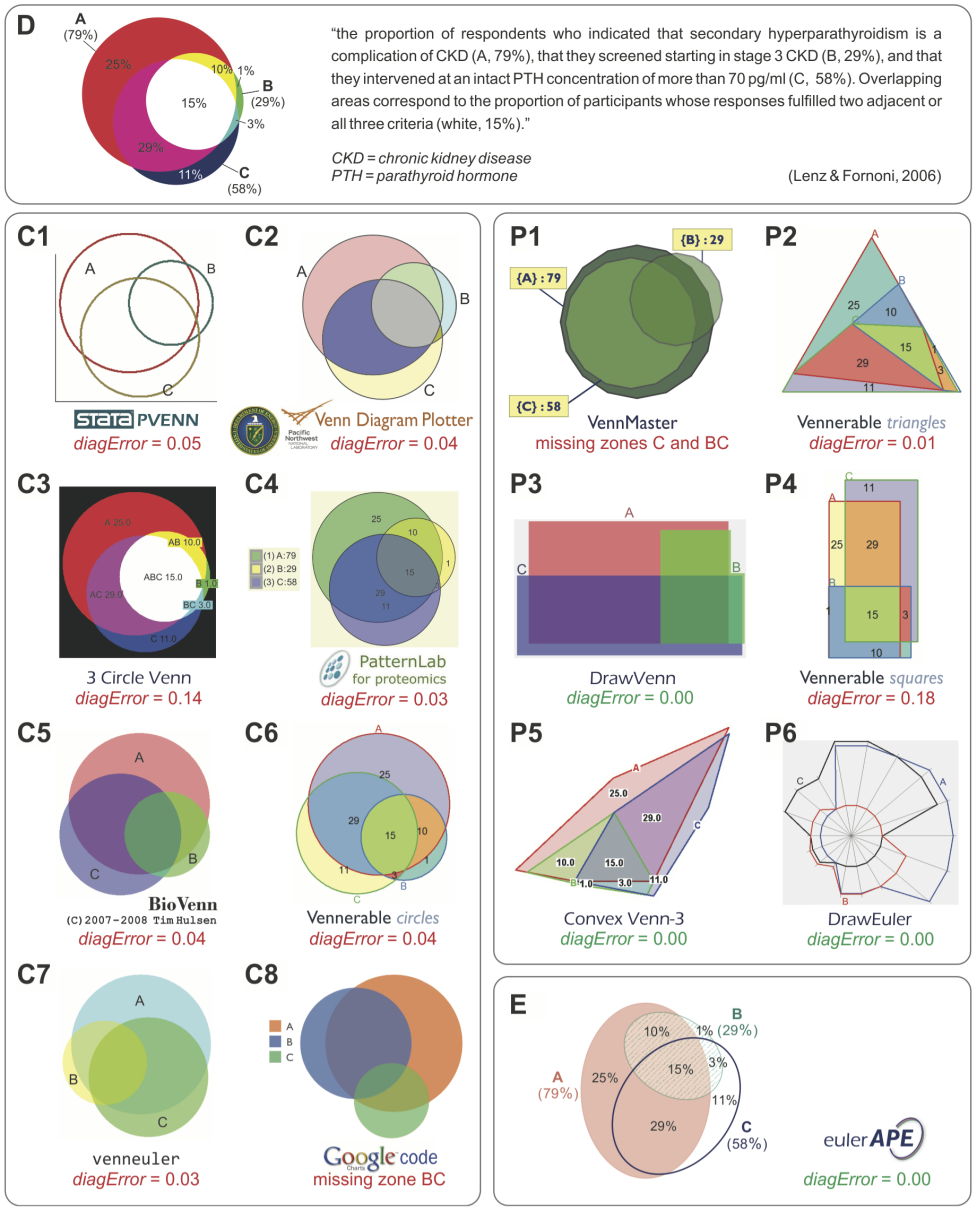
Diagrams generated by various drawing methods for the same medical data from a journal article. All the diagrams are meant to depict *ω* = {*A* = 0.25, *B* = 0.01, *C* = 0.11, *AB* = 0.10, *AC* = 0.29, *BC* = 0.03, *ABC* = 0.15}, which represents the findings of a medical survey from a journal article [37] that also included diagram D for *ω*. The diagrams generated for *ω* using circle-based drawing methods are marked as C, those of polygon-based methods are marked as P, and the only diagram with ellipses, that by euler*APE*, is E. Green indicates accurate diagrams with *diagError* ≤10^−6^. Red indicates diagrams with inaccurate or missing regions. D is a redrawing of Figure 5 (bottom) in [37], previously published under a CC BY license.

The first automatic drawing methods to use circles were developed for area-proportional Venn diagrams with two [5] and three [36] (known as 3 Circle Venn) curves. These were then used in areas such as medicine and health care (e.g., Figure 3-D [37] or Figure 3-C3). Various other methods were later developed. A few of these methods and examples of diagrams drawn using them, include: BioVenn [38] (e.g., Figure 3-C5, [39]); Venn Diagram Plotter [http://omics.pnl.gov/software/VennDiagramPlotter.php] (e.g., Figure 1C, Figure 3-C2, [13]); a module in PatternLab for proteomics [40] (e.g., Figure 3-C4, [41]); R packages, Vennerable [https://r-forge.r-project.org/projects/venerable] (e.g., Figure 3-C6, [42]) and venneuler [29] (e.g., Figure 3-C7, [43]); GeneSpring [http://www.strandgenomics.com/GeneSpring] (e.g., [44]); Google Venn Charts [https://developers.google.com/chart/image/docs/gallery/venn_charts] (e.g., Figure 3-C8); Stata's PVENN [http://ideas.repec.org/c/boc/bocode/s457368.html] (e.g., Figure 3-C1); SAS macro [45] (e.g., [27]); Matlab's VENN [http://www.mathworks.com/matlabcentral/fileexchange/22282-venn] and vennX [http://www.mathworks.com/matlabcentral/fileexchange/6116-proportional-venn-diagrams]; a web application [http://bioinforx.com/lims/cloud-based-free-research-tools-for-scientific-data-management-and-analysis/bxtoolbox] (e.g., [46]). Excluding venneuler, all of these methods draw area-proportional Venn diagrams with two or three circles and most are simple variants of the first devised method for three curves [36]. Various methods (e.g., BioVenn, PatternLab for proteomics) were specifically designed for biological data.

The latest proposed method, venneuler, is different from most others as it uses a statistical model for fitting an area-proportional diagram to the required quantitative data. The model is comprised of a normalized loss function *stress* (defined as the sum of squared residuals divided by the total sum of squares) and a mechanism to minimize the function. Compared to 3 Circle Venn [36] and a method VennMaster [47], [48] that draws the diagrams with convex regular polygons, venneuler is the most effective with respect to both accuracy and generation time. However, venneuler still generates inaccurate and misleading diagrams for most quantitative data due to the limitations of circles.

**Figure 4:**
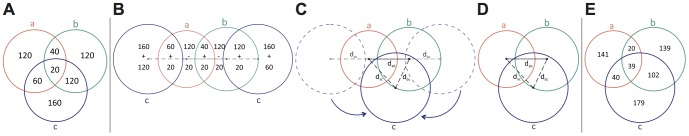
A method for constructing an area-proportional 3-Venn diagram using circles. (A) The quantitative values in each region indicate the required region areas, for which an area-proportional 3-Venn diagram should be drawn. (B) The first step of the construction whereby the three accurate 2-Venn diagrams are drawn. (C) The second step of the construction whereby the identical copies of the circle labelled *c* are rotated such that they overlap completely and only one circle labelled *c* is visible. (D) The instance when only three circles are visible, such that the regions of the 3-Venn diagram are obtained. (E) The actual area of the regions in the constructed diagram D, which, as in most cases when these diagrams are drawn with circles, do not correspond to the desired values in A. The numeric label in each region of this diagram indicates the regions' actual area.

An accurate area-proportional 2-Venn diagram can be drawn for any quantitative data using two circles *a* and *b*. This is fully constrained, as given *a* and *b* have radius *r_a_* and *r_b_* respectively and distance *d_ab_* between the centre of *a* and *b*, only one overlapping region area exists. When a circle *c* is added, the overlapping region of the 2-Venn diagram is split up into two and new regions are introduced. So a 3-Venn diagram is made up of three 2-Venn diagrams (i.e., *2-Venn_ab_*, *2-Venn_ac_*, *2-Venn_bc_*). Thus, an area-proportional 3-Venn diagram for the quantitative data indicated by the numeric labels in Figure 4A can be constructed by first drawing the accurate 2-Venn diagrams in Figure 4B (the numeric values in the regions indicate their current area), whereby two copies of circle *c* are drawn to ensure that both *2-Venn_ac_* and *2-Venn_bc_* are accurate. Then, rotate the left copy of *c* anticlockwise about the centre of *a* and the right copy of *c* clockwise about the centre of *b*. Only one circle *c* is required and thus, the two copies of *c* must be rotated until they overlap completely (Figure 4C). At this point, the overlapping regions in a 3-Venn diagram are automatically formed (Figure 4D). However, the resulting region areas (Figure 4E) are unlikely to be the same as those required (i.e., the numeric labels in Figure 4A) and yet, no changes can be made to improve the accuracy of a region area without making others less accurate.

### 2.2. Polygon-based

The first proposed method, VennMaster [47], [48], uses convex regular polygons. Such polygons are similar in shape to circles and thus the generated diagrams are often inaccurate (e.g., Figure 3-P1, [49]). Other methods use: triangles as in Vennerable [https://r-forge.r-project.org/projects/venerable] (e.g., Figure 3-P2); rectangles (e.g., [50]); orthogonal rectilinear curves as in DrawVenn [5] (e.g., Figure 1D, Figure 3-P3, [14]) and Vennerable [https://r-forge.r-project.org/projects/venerable] (e.g., Figure 3-P4); 4-sided and 5-sided convex polygons as in Convex Venn-3 [51] (e.g., Figure 1E, Figure 3-P5, [15]); parallelograms [52]; orthogonal polyominoes [53]; a combination of convex and non-convex, smooth and rectilinear curves as in VENNTURE [54] and in Vennerable [https://r-forge.r-project.org/projects/venerable]; convoluted polygons as in DrawEuler [55] (e.g., Figure 1F, Figure 3-P6, [16]) and Fan Diagrams [56]. A method that draws diagrams with polygons for any number of curves has been proposed but not implemented [57]. A recent method, Euler3, was devised to use polygons only when circles cannot be used [58].

## euler*APE* Method and Software

Our drawing method euler*APE* is based on the simple hill-climbing optimization technique to draw an area-proportional diagram with ellipses in a time that is relatively fast and that maintains users' attention. The area of the regions of the three intersecting ellipses is computed accurately and instantaneously using an analytic method that is based on integral calculus ([59] - Section 5.4).

Each of the quantities in the provided data, for which a diagram should be drawn, is first scaled by a factor of (*100* / *smallest quantity in the data*), so the same diagram is generated for quantitative data that is different but proportional. The scaled quantity corresponding to a region is then the required area of the region in the diagram to be generated. Later, the search for a solution that satisfies our diagram goodness measure (Section 3.1) commences, so that a diagram with region areas that are directly proportional to the scaled quantitative data is generated. A cost function directs the optimization process to a good solution (Section 3.2), in that, starting with a rational diagram for the required region areas (Section 3.3), the properties of the ellipses are adjusted based on the cost of the modified diagram (Section 3.4). The software is online and free to use (Section 3.5).

### 3.1. The Diagram Goodness Measure

To verify whether the region areas of an area-proportional diagram are accurately and directly proportional to the required quantitative data, euler*APE* uses the following measure:

If

- *ω* is the set of quantities for which a diagram had to be drawn,
- *d* is an area-proportional diagram generated for *ω*,
- *R* is the set of labels describing the required set of regions interior to the curves of the diagram,
- *ω* (*r*) 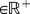 is the quantity assigned to *r* 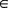 *R* that should be depicted by the area of *r* in *d*, and
- *A* (*r*) 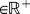 is the area of *r* 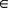 *R* in *d*,

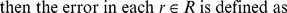





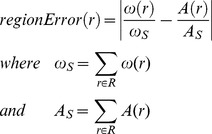
(1)and the error in *d* is defined as
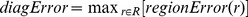
(2)so that *d* is a *good, accurate diagram with respect to ω*, in that its region areas are accurately and directly proportional to the quantities in *ω*, if and only if
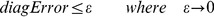
(3)


In euler*APE*, *ε* = 10^−6^, which value is consistent with that of other methods when defining a value for zero in their implementation (e.g., venneuler [29]). The value of *regionError* and *diagError* is always in [0,1].

Rather than using the absolute region area, euler*APE*'s measure considers the proportion of each region area to the area of the total diagram. An inaccuracy in one region could make other accurate regions or curves in the diagram seem erroneous, as regions and curves might be compared for their area to be estimated. This can be avoided by considering the area of the region with respect to the total area of the diagram. A similar measure to the one used by euler*APE* was considered by a previous drawing method [36].

### 3.2. The Cost Function

To obtain a good, accurate diagram with respect to the required quantitative data (as defined in Section 3.1), our optimization algorithm minimizes a cost function that takes into account the accuracy of the diagram as well as paths that could lead to a local minimum. In an informal experimentation, we observed that the cost function of most of the current methods, such as venneuler's *stress* [29] and Chow and Rodgers's ‘idealistic’ function [36], often drive the optimization to a local minimum, as the overall error of the diagram is reduced at the expense of diminishing a region to a point where it is close to non-existent and its actual-to-required area ratio is close to zero. In such cases, no further changes can be carried out otherwise the diagram would no longer depict all of the required regions. Following our observation, we devised the following novel cost function:

If

- *ω* is the set of quantities for which a diagram should be drawn,
- *ω′* is the set of scaled quantities of *ω* (obtained as explained earlier in Section ‘euler*APE Method and Software*’), indicating the required region areas in the required good diagram for *ω*,
- *d* is an area-proportional diagram that is explored for *ω* during the optimization,
- *R* is the set of labels describing the required set of regions interior to the curves of the diagram,
- *ω′* (*r*) 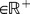 is the area that *r* 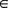 *R* should have in the required good diagram, and
- *A* (*r*) 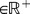 is the area of *r* 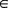 *R* in *d*,

then the cost of *d* is defined as 
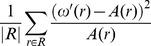
(4)


Thus, the cost of a diagram is the mean of the cost of all the regions in that diagram. The sum could have been used since this work focuses on 3-Venn diagrams. However, we used mean so this function could be used in other future algorithms for diagrams with any number of curves and overlaps.

A diagram is generated for the required region areas scaled (i.e., those in *ω′*) and so, it is adequate to consider the absolute area of the regions. A similar approach was adopted by previous techniques, such as VennMaster [47], [48]. If relative region areas are considered as in our goodness measure, the search could be restricted, particularly at the initial stages of the optimization when a good but non-refined solution is adequate.

The denominator *A*(*r*) for *r* 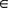 *R* in our function prevents the optimization from taking paths that reduce the overall error of the diagram at the expense of diminishing the actual-to-required area ratio of a region, leading to a local minimum. With our function, a region with a very small actual-to-required area ratio will have a very large cost and thus prevent the optimization from taking such paths.

Though our cost function in Equation (4) is non-dimensionless, it is still adequate as the provided quantitative data is scaled before a diagram is generated for this data. However, we still considered ways how to make Equation (4) dimensionless. The denominator *A*(*r*) for *r* 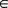 *R* could be squared as in 
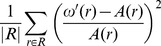
(5)or the numerator could be the absolute difference between the required and actual region area not squared as in
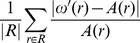
(6)


However, these two dimensionless functions will have a different effect from that intended by our non-dimensionless cost function, as the cost of a region would be much smaller than that in Equation (4) when the actual region area is greater than one and much greater than that in Equation (4) when the actual region area is less than one. This means that large errors would be less easily identified and the actual region area would have a greater impact on the cost than that intended to identify regions with a very small actual-to-required area ratio. The cost of a region in Equation (6) is the same as that in Equation (5) except that it is not squared. Thus, if large errors in a region result in a smaller cost in Equation (5) than in Equation (4), then in Equation (6) the cost would be even smaller, making it more difficult to identify inappropriate paths. So, we expect Equation (5) and Equation (6) to be less effective than Equation (4), and for Equation (6) to perform worse than Equation (5).

To choose the most effective cost function for euler*APE*, we conducted an experimental comparison of the following cost functions: **F1**, venneuler's *stress*, using the source code of venneuler version 1.1-0, but with *ω′* (*r*) and *A*(*r*) for the required and the actual area of a region *r* 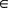 *R*; **F2**, the first component of Chow and Rodgers's ‘idealistic’ function, which is related to our *regionError*; **F3**, the sum of the squared relative error of the regions; **F4**, the maximum of the relative error of the regions; **F5**, the sum of the relative error of the regions; **F6**, our non-dimensionless cost function Equation (4); **F7**, our dimensionless cost function Equation (5); **F8**, our dimensionless cost function Equation (6).

The cost function of the optimization algorithm in Section 3.4 was replaced by one of F1–F8 and used to generate diagrams (with the rerun option disabled) for two libraries of 10,000 random 3-set data items each: **L1** with quantitative data for which an accurate 3-Venn diagram with ellipses is known to exist; **L2** with quantitative data for which it is not clear whether an accurate 3-Venn diagram with ellipses can be drawn. The details and results of this evaluation are available in Micallef's PhD thesis [59] (Chapter 5 and Appendix A).

This evaluation and experimental comparison indicated that our non-dimensionless cost function F6 is the most effective in:

- Generating good diagrams for quantitative data for which a good diagram is known to exist;
- Converging to diagrams that have a low *diagError* even when a good diagram cannot be drawn with respect to the given data;
- Identifying and avoiding paths that lead the optimization to a local minimum when the overall error of the diagram is reduced at the expense of diminishing the area of a region to a point where it is close to non-existent and its actual-to-required region area ratio is close to zero;
- Taking the least amount of the time and iterations to generate a diagram, particularly for data for which a good diagram is known to exist;
- Generating a large majority of the diagrams (97.3%, *N* = 20000) within a time (1 second) that ensures that the users' train of thought is maintained, and generating nearly all the diagrams (99.6%, *N* = 20000) within a time (10 seconds) that ensures that the users' attention is maintained.

The effectiveness of F6 over the other cost functions with respect to the generation of good diagrams, the *diagError* of the non-good diagrams, generation time and number of iterations was highly evident for the diagrams generated for the data in L1, but less evident for those of L2. The results for L1 could be more important than those for L2, as an accurate diagram with ellipses exists for all of the 10,000 data items in L1. There is 3-set data for which an accurate diagram cannot be drawn with convex curves [30] (and thus ellipses) and it is unknown how much of this data is in L2. This evaluation also demonstrated that all of the cost functions (F1–F5), except for those we devised (F6–F8), often direct the optimization to a local minimum as the actual-to-required area ratio of a region is reduced to a value close to zero. Thus the cost function should heavily weight regions whose area is very small with respect to that required, as done in F6–F8. However, as expected, our dimensionless cost functions F7 and F8 were not as effective as our non-dimensionless F6.

Following the results of this evaluation, euler*APE* uses our cost function F6, as given in Equation (4).

### 3.3. The Starting Diagram

The optimization process has to commence with a solution. This is often an arbitrary or an invariant solution. Both types of starting diagrams were considered for euler*APE*. The arbitrary starting diagram was a Venn diagram comprised of three ellipses whose properties were assigned random values. The invariant starting diagram was a Venn diagram with 3-fold rotational symmetry, comprised of three equally-sized circles and regions that were similar in size, except for those in only one curve that were around three times as much as the rest. As expected, both types of starting diagrams led to poor results in terms of generation time and diagram quality, as such starting diagrams do not take into account the data that the good solution must satisfy and are more likely to direct the optimization to a local minimum [60], [61]. We wanted euler*APE* to be deterministic and so an arbitrary starting diagram was particularly inappropriate, as different diagrams would be generated for the same data every time the optimization is run (as in e.g., VennMaster [47], [48]).

A rational starting diagram that is adapted to the required quantitative data is more effective, as it reduces convergence time and the likelihood of reaching a local minimum. Such a starting diagram is used by for instance venneuler [29]. The starting diagram used and generated by euler*APE* is drawn using three ellipses with equal semi-axes, so the ellipses are depicted as circles. An angle of rotation of 0, π/3 and 2π/3 is assigned to the three respective ellipses to ensure that the entire space of possible angles of rotation is considered during the optimization. Assigning an appropriate centre for the ellipses is difficult and yet important as this determines the accuracy of the region areas. The two largest required ellipses, *e_1_* and *e_2_*, in the diagram are chosen and an area-proportional 2-Venn diagram that accurately depicts the data corresponding to *e_1_* and *e_2_* and their overlap is drawn using Chow and Ruskey's bisection method [5]. This increases the likelihood that the overall starting diagram is close to that required, as an accurate area-proportional Venn diagram can be drawn with circles for any data with two sets [5] and the 2-Venn diagram with *e_1_* and *e_2_* covers a large portion of the starting diagram.

**Figure 5:**
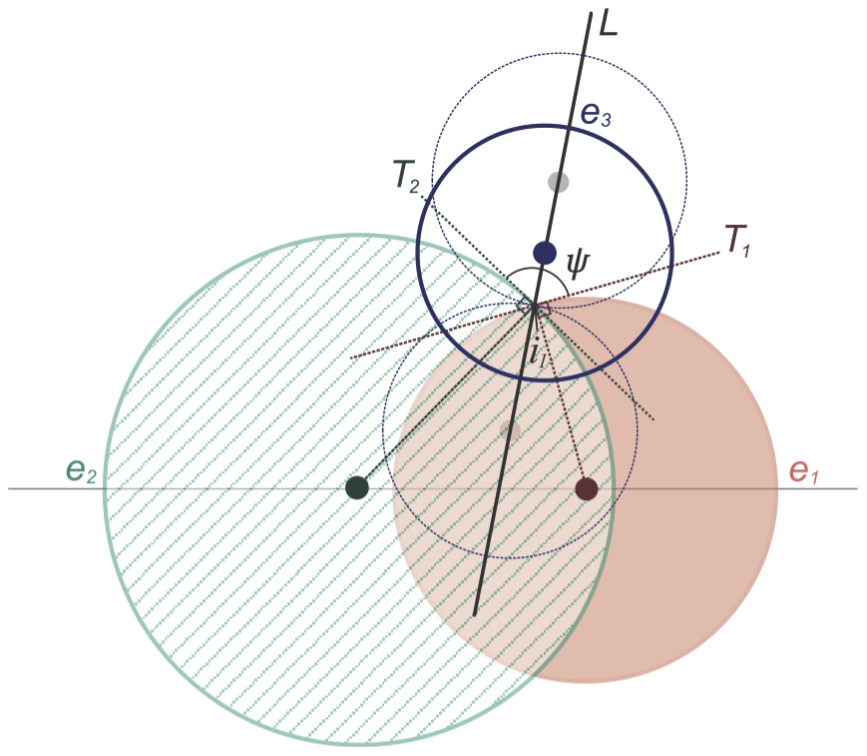
The starting diagram generator bisecting the interval along bisector line to position the third ellipse. The centre of ellipse *e*_3_ is a point on the line *L* bisecting the angle *ψ* between the two tangents *T*_1_ and *T*_2_. The bisection method is applied in the interval indicated by the faded blue circles along *L*. The obtained centre should minimize the discrepancy of the required and the actual area of the region in exactly the three ellipses.

Changes to the ellipses during the optimization affect the area of the region in exactly the three ellipses. So a starting diagram that minimizes the error of this region seems helpful. To achieve this, the centre for the third ellipse *e_3_* is obtained by applying the bisection method in an interval along a line *L*, as shown in Figure 5. *L* is the bisector of the angle, *ψ*, between two lines, *T_1_* and *T_2_*, that are respectively tangents to *e_1_* and *e_2_* at *i_1_* (i.e., the upper intersection point of *e_1_* and *e_2_*). The interval along *L* is (*u*, *l*) where *u* is a point that lies above another point *l* on *L*, such that, as shown by the faded blue circles in Figure 5, the centre of *e_3_* must be between *u* and *l* (but not equal to *u* or *l*) for *e_3_* to intersect each of *e_1_* and *e_2_* twice and form the seven regions interior to the curves of a 3-Venn diagram. As illustrated by the faded blue circles in Figure 5, the endpoints of the interval as well as any value that is not in the interval will generate a diagram that is not a Venn diagram.

Out of the starting diagrams generated for 10,000 random 3-set data items for which an accurate Venn diagram with ellipses is known to exist, 63% had *diagError* ≤0.05 (i.e., 5%). The value of *diagError* is always in [0,1] and so, this result indicates that the generated starting diagrams are relatively close to the required solution. Also, the time to generate the diagrams is 10 times less than the 0.1 second limit for an instantaneous response [62], [63] with a mean of 8 milliseconds for the 10,000 diagrams.

### 3.4. The Optimization Algorithm

Our simple hill-climbing algorithm commences with a rational starting diagram and systematically adjusts the properties of its ellipses to minimize our cost function, until a good diagram with respect to the given quantitative data is obtained. Though simple and a local search, it rarely encounters a local minimum and if it does, our algorithm is capable of handling such cases and obtain a good solution whenever an accurate area-proportional 3-Venn diagram drawn with ellipses is known to exist for the given data (as shown in Section 4.1).

**Figure 6:**
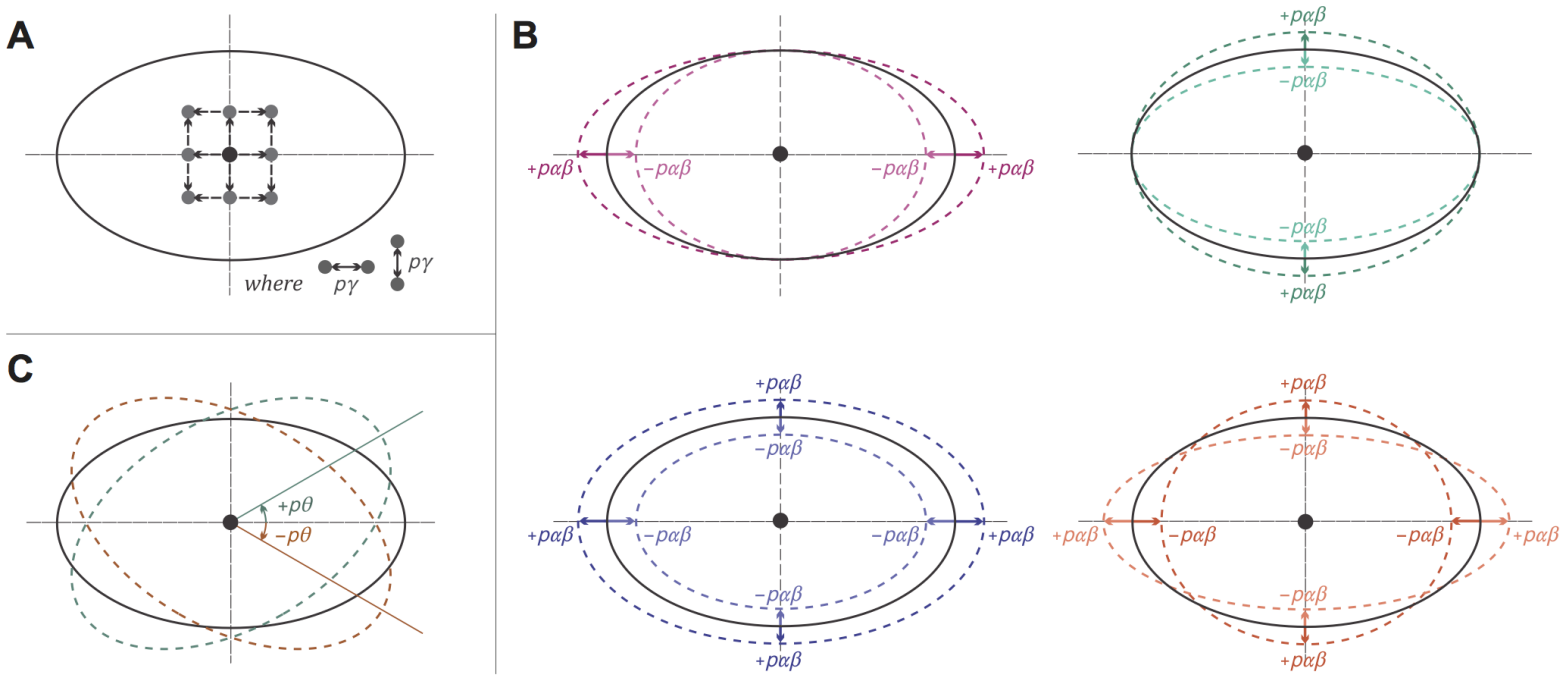
The different ways the ellipses' properties are modified during the optimization search process. At every iteration of the optimization algorithm, the (A) centre, (B) semi-axes and (C) angle of rotation of every ellipse are respectively modified by parameters *pγ*, *pαβ* and *pθ* in search for other solutions. (A) The grey points indicate the new centres that are obtained when one or both coordinates of the centre of an ellipse (solid black) are increasing or decreasing by *pγ*. (B) A label *+pαβ* means that that semi-axis was increased by the *pαβ* percentage, while -*pαβ* means that that semi-axis was decreased by the *pαβ* percentage. The dashed ellipses indicate how an ellipse (solid black) is changed when: (top, left) only the semi-major axis is increased or decreased by *pαβ*; (top, right) only the semi-minor is increased or decreased by *pαβ*; (bottom, left) the semi-axes are both increased or both decreased by *pαβ*; (bottom, right) one of the semi-axes is increased and the other is decreased by *pαβ*. (C) The dashed ellipses indicate how an ellipse (solid black) is changed when its angle of rotation is increased or decreased by *pθ*.

Our optimization algorithm is characterized by the following three parameters that determine how at every iteration, each ellipse *e* is modified in search for other possible solutions:

- *pγ*, the number of pixels by which one or both coordinates of the centre of *e* are modified to explore eight new centres for *e*—these are shown in Figure 6A, where the black ellipse and black point are *e* and its centre prior to any change, and the grey points are the eight new centres for *e*;
- *pαβ*, the scaling percentage by which one or both semi-axes of *e* are modified to explore eight new semi-axes for *e*—these are shown in Figure 6B, where the solid black ellipse is *e* prior to any change and the dashed coloured ellipses are *e* with the eight new semi-axes;
- *pθ*, the number of radians by which the angle of rotation of *e* is modified to explore two new angles of rotation for *e*—these are shown in Figure 6C, where the solid black ellipse is *e* prior to any change and the dashed coloured ellipses are *e* with the two new angles of rotation.

Changes that lead to a reduced cost of the diagram are accepted. At the start, *pγ* = 10 pixels, *pαβ* = 5% and *pθ* = 2π/3. These values were chosen after the diagram generation for different 3-set data was observed for different *pγ*, *pαβ* and *pθ* values. If, at the end of an iteration, a property of the ellipses is not changed, the value of the respective parameter is reduced linearly (halved). This means that major changes only occur at the start when the search space is explored for a good yet non-refined solution. As the values of *pγ*, *pαβ* and *pθ* are reduced further, minor changes to the diagram are explored, so that the diagram is refined to the required good solution. This cooling schedule, similar to that used in the global optimization method of simulated annealing, reduces the likelihood of converging to a local minimum and the time required to converge to the good solution. We halve the parameters as we observed that faster cooling rates restrict diagram refinement, while slower cooling rates are computationally expensive. This is the algorithm for the diagram generation process given a set of quantities, *ω*:

**Algorithm** euler*APE* (*ω*)

**Input**: *ω* is a set of seven quantities each corresponding to a region interior to the curves of a 3-Venn diagram

**Output**: an area-proportional 3-Venn diagram drawn with ellipses for *ω* and whether the diagram is accurate

1: *d* ← a rational starting diagram with respect to *ω*

2: **if** *d* is a good diagram for *ω* by Equation (3) **then**

3:   **return** *d*, *accurate*

4: **end if**

5: *ω′* ← the set of scaled quantities of *ω*

6: *pγ* ←10 pixels, *pαβ* ← 5%, *pθ* ← 2π/3

7: **do**

8:   **for** every ellipse *e* in *d* **do**

9:      *centres* ← the eight centres obtained by *pγ* for *e*

10:     **for** each *c* in *centres* **do**

11:       **if** the cost of *d* by Equation (4) is reduced when the centre of *e* in *d* is *c* **then**

12:         Change the centre of *e* in *d* to *c*

13:       **end if**

14:     **end for**

15:     *semiaxes* ← the eight semi-axes obtained by *pαβ* for *e*

16:     **for** each *s* in *semiaxes* **do**

17:       **if** the cost of *d* by Equation (4) is reduced when the semi-axes of *e* in *d* are *s* **then**

18:         Change the semi-axes of *e* in *d* to *s*

19:       **end if**

20:     **end for**

21:     *rotations* ← the two angles of rotation obtained by *pθ* for *e*

22:     **for** each *r* in *rotations* **do**

23:       **if** the cost of *d* by Equation (4) is reduced when the angle of rotation of *e* in *d* is *r* **then**

24:         Change the angle of rotation of *e* in *d* to *r*

25:       **end if**

26:     **end for**

27:   **end for**

28:   **if** no ellipse in *d* had its centre changed **then**

29:     Divide *pγ* by 2

30:   **end if**

31:   **if** no ellipse in *d* had any of its semi-axes changed **then**

32:     Divide *pαβ* by 2

33:   **end if**

34:   **if** no ellipse in *d* had its angle of rotation changed **then**

35:     Divide *pθ* by 2

36:   **end if**

37:   **if** *pγ* ≤*ε*, *pαβ* ≤*ε* and *pθ* ≤*ε*, where *ε* = 10^−6^ **then**

38:     **return** *d*, *inaccurate*

39:   **else**

40:     **if** *d* is a good diagram for *ω* by Equation (3) **then**

41:       **return** *d*, *accurate*

42:     **end if**

43:   **end if**

44: **loop**

Step 38 is reached when a local minimum is encountered. To handle such cases, euler*APE* has a rerun option which when enabled, it reruns the optimization using starting values for *pγ*, *pαβ* and *pθ* that are 20% larger than those used in the previous run. euler*APE* then terminates either when a good diagram is found or when 10 reruns are completed and a good diagram is yet not found. In the latter case, the diagram with the lowest *diagError* out of the 11 generated diagrams is returned. We opted for a 20% increase in the parameter values following our information experimentation of different parameter values when we observed cases that could benefit from such an increase and extended exploration of the search space.

### 3.5. Availability and How to Use

The software executable and the Java source code are freely available under the GNU General Public License version 3 at www.eulerdiagrams.org/eulerAPE. The latest release of euler*APE* should be downloaded from the webpage and opened by clicking on the downloaded jar file. Three steps are then required to use euler*APE*:

1. *enter* the quantities to be depicted by the regions of the diagram—these quantities should be typed in manually, generated randomly or loaded from a file;
2. *select* preferences—including: whether the diagram should be saved, how the diagram should be displayed (e.g., labels, colours, ellipses or circles), whether to view the search process;
3. *generate* the diagram—by clicking on the ‘RUN’ button.

Further details, example how to load the required quantitative data from a file or how to save the diagram, are available on euler*APE*'s webpage. The latest release, v3.0.0, has been fully tested on Windows and Mac OS X, is locale-independent, supports command-line execution (details on euler*APE*'s webpage), and exports diagrams in png and svg format as well as in textual format with details about the properties of the ellipses in the diagram.

## Effectiveness of euler*APE* and Ellipses

To evaluate the effectiveness of ellipses in drawing accurate area-proportional 3-Venn diagram for given data, we first evaluated the effectiveness of euler*APE* in drawing good diagrams for drawable 3-set data, that is data for which a good diagram is known to exist (Section 4.1). Being able to handle such data means that euler*APE* can avoid and handle local minima and if euler*APE* cannot draw an accurate, good diagram for any random data in our second evaluation (Section 4.2), then it is highly likely that a good diagram drawn with ellipses does not exist for that data. In this way, we were able to identify characteristics of 3-set data that are drawable with ellipses (Section 4.2). In our second evaluation, we also generated diagrams for the same random data using a variant of euler*APE* that restricts the ellipses to circles to identify whether in these cases an accurate diagram could be drawn with circles (Section 4.2). We then compared these results to the diagrams generated by the latest circle-based method, venneuler [29], for the same random data (Section 4.3). Finally, we compared the accuracy and the curve aesthetics of the diagrams generated by euler*APE* and various other drawing methods using circles or polygons for real world data in a medical application area (Section 4.4).

The error of the diagrams generated by euler*APE* and other drawing methods was measured by *diagError* in Equation (2) whose value is in [0,1]. Good diagrams are those that satisfy our diagram goodness measure in Equation (3) and are thus diagrams that depict all the required regions and have *diagError* ≤10^−6^. In our experiments, the number of iterations and the time taken to generate the diagrams were also recorded.

This evaluation focuses on 3-set data that associates a quantity greater than zero to each of the seven regions interior to the curves of a 3-Venn diagram. Diagrams with region areas that are zero percent of the total area of the diagram can still be drawn with euler*APE*, but further evaluation is required in the future.

In this section, **L1** and **L2** refer to two libraries each with 10,000 sets of seven numbers greater than zero. The numbers of a set in L1 are the region areas of a 3-Venn diagram generated after random values are assigned to the properties of three overlapping ellipses. The numbers of a set in L2 are randomly obtained from a uniform distribution in the interval [1,10000]. The data in these two libraries is different from that used in the evaluation of different cost functions in Section 3.2.

### 4.1. For Drawable Data

Diagrams were generated with ellipses by euler*APE* for the 10,000 drawable data items in L1. The rerun option of the optimization algorithm (Section 3.4) was enabled to verify whether euler*APE* still draws a good diagram if a local minimum is reached in the first run.

**Figure 7:**
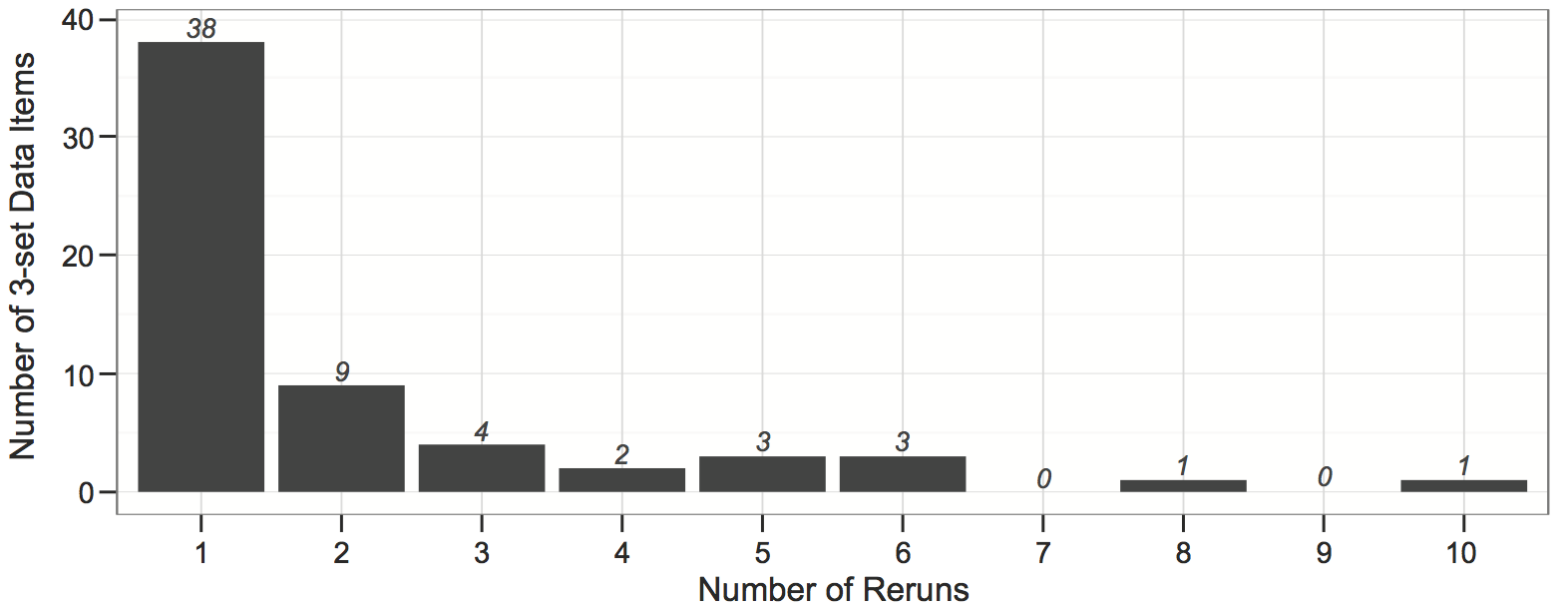
The number of reruns to generate a good diagram for 61 data items in L1. The number of reruns (1–10) that were required for euler*APE* to generate a good diagram for the 61 data items in L1 for which a non-good diagram was generated during the first run.

By the first run, good diagrams were generated for 9939 of the 10,000 data items (i.e., 99.4%). Despite generating a non-good diagram for the remaining 61 data items (i.e., 0.6%), the *diagError* of these diagrams was relatively low (median 1.06×10^−4^, mean 2.38×10^−3^, minimum 1.02×10^−6^, maximum 3.09×10^−2^) and 54 of them (i.e., 88.5%) had *diagError* ≤0.01. Good diagrams were generated for all of these 61 data items after the optimization algorithm was rerun. For the majority (38/61, i.e., 62.3%), a good diagram was generated after the first rerun (Figure 7; number of reruns, median 1 and mean 2.1). Thus, with 99% confidence, these results indicate that for 99.2% to 99.6% of drawable 3-set data, euler*APE* draws a good diagram during the first run, and for 99.9% to 100.0% of the same type of 3-set data, euler*APE* draws a good diagram after one to 10 reruns.

**Figure 8:**
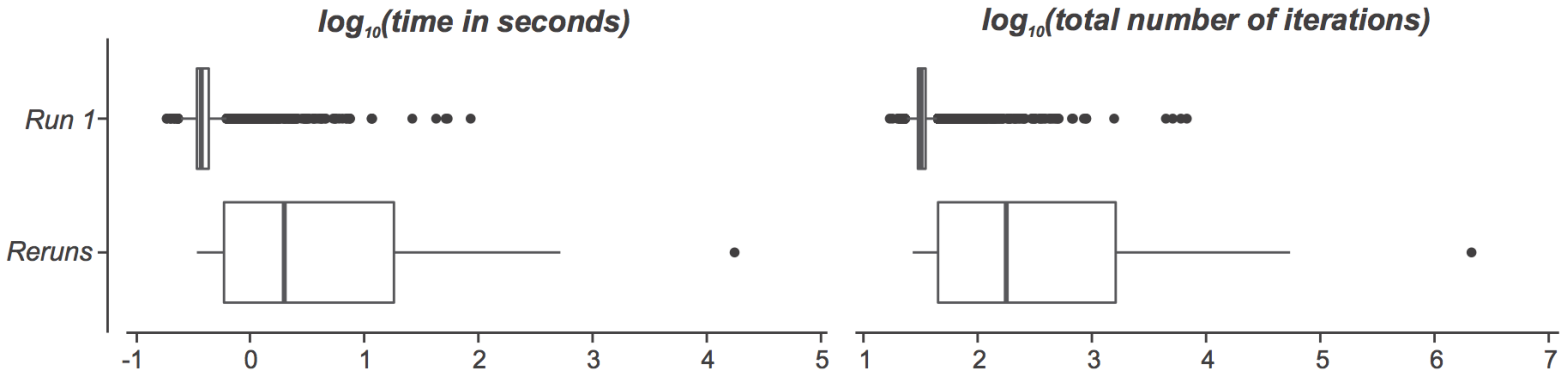
Time and total number of iterations to generate good diagrams for data in L1. The *log_10_* (*time in seconds*) and *log_10_*(*total number of iterations*) taken to generate good diagrams for 9939 of the 10,000 data items in L1 during the first run (labelled as ‘*Run 1*’) and for 61 of the 10,000 data items in L1 during any of the one to a maximum of 10 reruns (labelled as ‘*Reruns*’).

When the optimization algorithm is rerun, more time and total number of iterations are required to generate a good diagram (Figure 8). Even so, the generation of the 10,000 good diagrams had an overall median and mean time of respectively 0.4 seconds and 2.5 seconds, and an overall median and mean number of iterations of respectively 32 and 273. Also, for 97.7% of the 10,000 data items, a good diagram was generated within 1 second (98.1% and 34.4% of respectively the 9939 good diagrams generated during first run and the 61 good diagrams generated during a rerun), and for 99.7% of the 10,000 data items a good diagram was generated within 10 seconds (99.9% and 62.3% of respectively the 9939 good diagrams generated during first run and the 61 good diagrams generated during a rerun). So, with 99% confidence, these results indicate that for 97.4% to 98.0% of drawable 3-set data, euler*APE* draws a good diagram within 1 second, and for 99.6% to 99.8% of the same type of 3-set data, euler*APE* draws a good diagram within 10 seconds. These results are important as a response time of 1 second ensures the users' train of thought is uninterrupted and a response time of 10 seconds ensures the users' attention is retained [62], [63].

**Figure 9:**
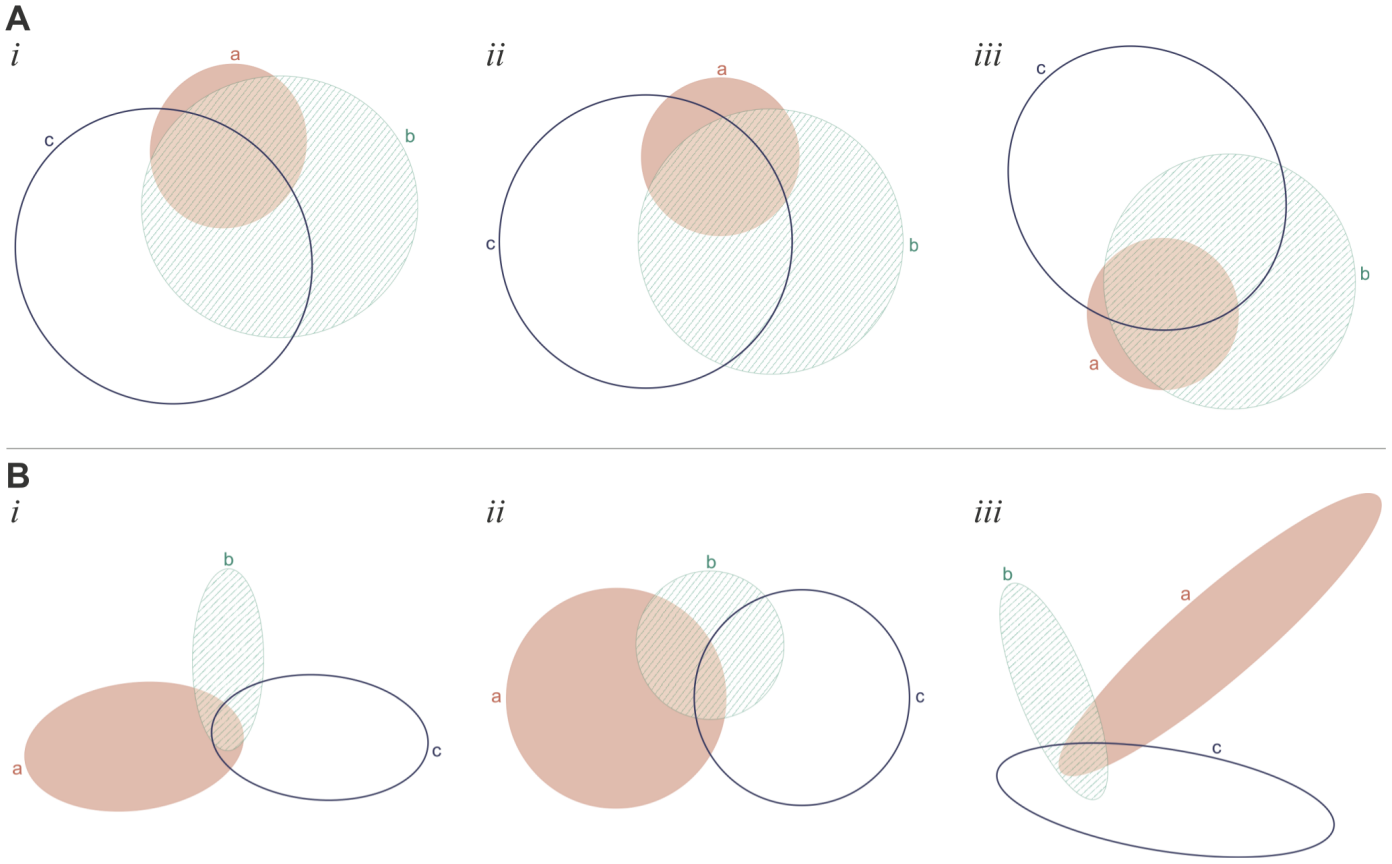
Examples of good diagrams generated after the first run for data in L1. (A) and (B) illustrate (*i*) the good diagram that was found using (*ii*) the starting diagram generated for the data item in L1 ({*a* = 2273, *b* = 24458, *c* = 44454, *ab* = 7116, *ac* = 740, *bc* = 18807, *abc* = 12092} for A and {*a* = 17033, *b* = 6248, *c* = 16230, *ab* = 615, *ac* = 289, *bc* = 840, *abc* = 922} for B) that was equal to the set of region areas of (*iii*) a randomly generated 3-Venn diagram.

Figure 9A and Figure 9B illustrate (*i*) the good diagram obtained from (*ii*) the starting diagram generated for the data item in L1 that was equal to the set of region areas of (*iii*) a randomly generated diagram. These examples illustrate that whenever possible euler*APE* draws circle-like curves (e.g., the semi-axes of ellipses *a*, *b* and *c* in Figure 9A *i* differ by 6.0%, 5.4% and 8% respectively). In other cases, elongated ellipses are required to accurately draw the desired region areas (e.g., in Figure 9B *i*, the required area for the regions located in only one of the curves is large compared to that of other regions), but the curves are still highly symmetric and distinguishable in shape from the regions, thus facilitating diagram comprehension [32]. Also, the curves in the diagrams are often evenly distributed, thus increasing the likelihood that the curves are easily distinguishable.

**Figure 10:**
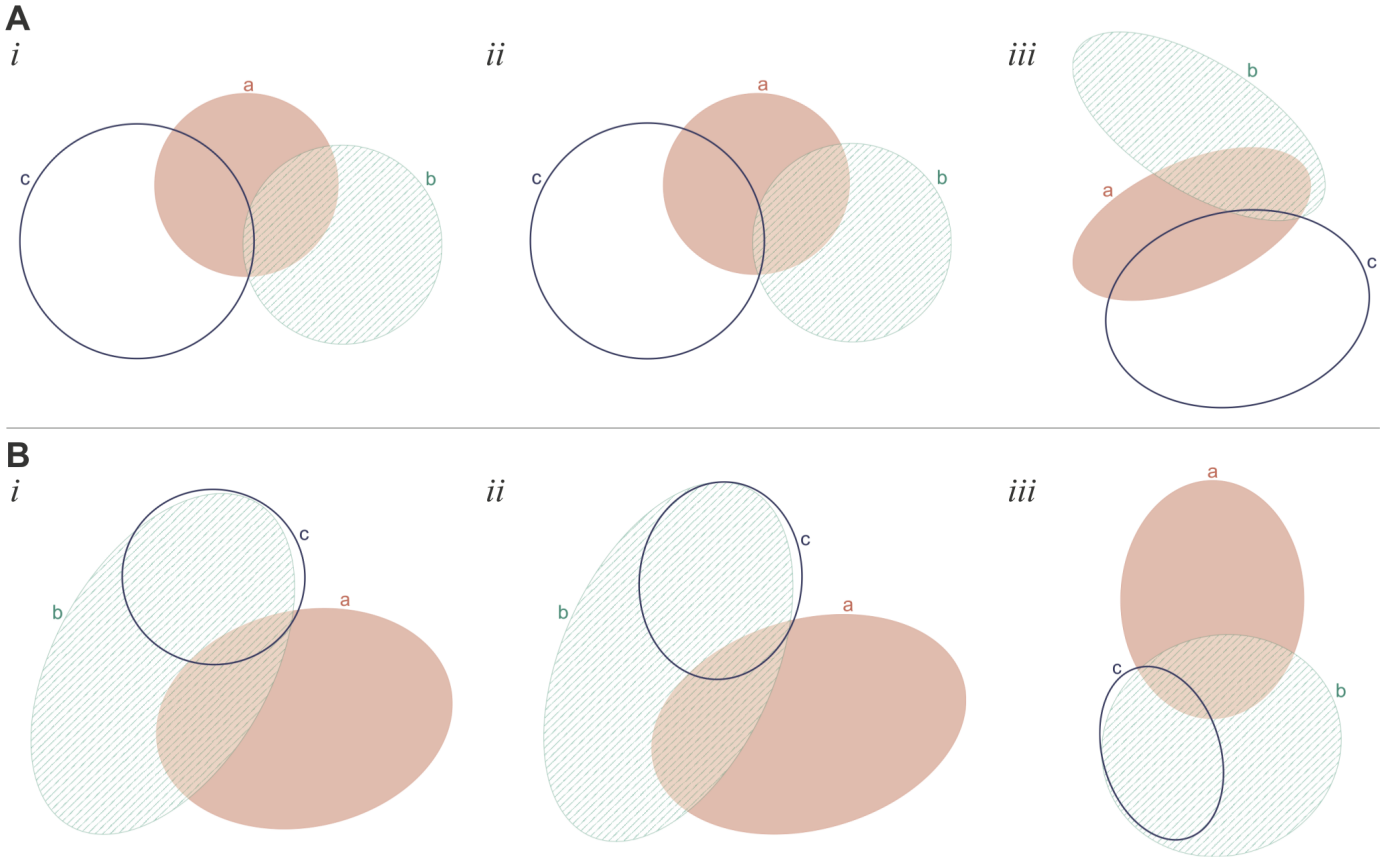
Examples of good diagrams generated after the first rerun for data in L1. (A) An example of (*i*) a non-good diagram with a very low *diagError* (6.51×10^−4^) generated during the first run and (*ii*) the good diagram generated during the first rerun for the data ({*a* = 10018, *b* = 27132, *c* = 39737, *ab* = 9567, *ac* = 11454, *bc* = 3, *abc* = 668}) in L1 obtained from (*iii*) a random diagram. The good diagram in *ii* was generated in 1.2 seconds and 86 iterations (including the first run and the one rerun). (B) An example of (*i*) a non-good diagram with a low *diagError* (8.38×10^−3^) generated during the first run and (*ii*) the good diagram generated during the first rerun for the data ({*a* = 53804, *b* = 39550, *c* = 1256, *ab* = 15606, *ac* = 15, *bc* = 29904, *abc* = 3597}) in L1 obtained from (*iii*) a random diagram. The good diagram in *ii* was generated in 2.9 seconds and 367 iterations (including the first run and the one rerun).

The majority of the non-good diagrams generated during the first run had a low *diagError* and just needed further refinement. Figure 10A *i* is an example of such a diagram generated during the first run with *diagError*  = 6.51×10^−4^. Figure 10A *ii* is the good diagram that was generated after one rerun for the data in L1 obtained from the diagram in Figure 10A *iii*. The area of region *bc* is 0.003% of the area of the total diagram and yet euler*APE* was still capable of accurately computing the region areas and generate a good diagram. Figure 10B *ii* is an example of a good diagram that was generated after the first rerun for the data in L1 obtained from the diagram in Figure 10B *iii*. As shown in Figure 10B *i*, during the first run, the optimization was trapped in a local minimum as ellipse *b* approached the edge of ellipse *c* (making region *c* seem like it was made up of two regions). By rerunning the optimization algorithm, different paths were explored and Figure 10B *ii* was generated.

The results of this evaluation indicate the effectiveness of euler*APE* in drawing good diagrams for drawable data. So, if euler*APE* cannot draw a good 3-Venn diagram for a set of seven quantities greater than zero, each corresponding to a region in the diagram, then it is highly likely that a good 3-Venn diagram drawn with ellipses does not exist for that quantitative data.

### 4.2. For Random Data

Since euler*APE* can generate good diagrams for drawable data (Section 4.1), we used euler*APE* to evaluate the effectiveness of ellipses in drawing accurate area-proportional 3-Venn diagrams for any random 3-set data with values greater than zero. Diagrams for the 10,000 data items in L2 were generated using euler*APE*. The data in L2 is made up of random values and thus, it is unknown whether an accurate diagram drawn with ellipses exists for this data (i.e., whether the data is drawable). The rerun option of the optimization algorithm (Section 3.4) was enabled to ensure that a good diagram is drawn for all drawable data. Diagrams with circles are preferred and the most effective [32], so we also evaluated whether an accurate, good diagram can be drawn using a variant of euler*APE* that restricts the ellipses to circles for any of the 10,000 data items in L2.

Good diagrams drawn with ellipses were generated for 8607 of the 10,000 data items in L2 (i.e., 86.1%)—8372 after the first run (i.e., 97.3% of the 8607) and 235 after one to a maximum of 10 reruns (i.e., 2.7% of 8607). More than half of the 235 good diagrams (56.2%) were generated during the first rerun and only one was generated after 10 reruns, as the *diagError* of the non-good diagrams generated for these data items during the first run was relatively low (*diagError* in [1.51×10^−6^, 3.28×10^−2^] with median 1.89×10^−3^ and mean 3.77×10^−3^).

None of the diagrams drawn with circles for the 10,000 data items in L2 were good, and the *diagError* of these diagrams was greater than that of the non-good diagrams drawn with ellipses (median, mean: 6.28×10^−2^, 6.73×10^−2^ for circles; 1.65×10^−2^, 2.11×10^−2^ for ellipses). With a 99% confidence, these results indicate that for 85.2% to 86.9% of random 3-set data, a good diagram can be drawn (using euler*APE*) with ellipses, and for 0.0% to 0.1% of random 3-set data, a good diagram can be drawn (using euler*APE*) with circles. There are 3-set data for which an area-proportional 3-Venn diagram cannot be drawn accurately using convex curves [30] and so, drawing good diagrams with ellipses for a large majority of the 10,000 random data items in L2 indicates great potential for using curves that are regular and smooth as circles, but more general and with more degrees of freedom like ellipses.

The time and number of iterations that were required for the generation of the good diagrams using ellipses were similar to those of our evaluation in Section 4.1 (this evaluation: medians 0.4 seconds and 35 iterations, means 1.9 seconds and 201 iterations, *N* = 8607). Non-good diagrams with ellipses required more time and iterations as the optimization algorithm was rerun a maximum of 10 times (medians, 4.0 seconds, 586 iterations; means, 25.9 seconds, 4417 iterations). Similarly, the diagrams drawn with circles required more time and iterations (medians, 3.2 seconds, 500 iterations; means 3.4 seconds, 529 iterations), as none were good.

The majority of the 10,000 diagrams with ellipses were generated within 1 second (84.1%—8405/8607 good, 0/1393 non-good) and nearly all with ellipses within 10 seconds (96.9%—8569/8607 good, 1119/1393 non-good). So similar to Section 4.1, with 99% confidence, these results indicate that for 83.1% to 85.0% of random 3-set data, euler*APE* draws a diagram with ellipses within 1 second, and for 96.4% to 97.3% of the same type of 3-set data, euler*APE* draws a diagram with ellipses within 10 seconds. Out of the 10,000 diagrams with circles, none were generated within 1 second, but 99.6% (9959/10,000) were generated within 10 seconds.

This evaluation also revealed that data for which an area-proportional 3-Venn diagram can be drawn with ellipses often has larger areas for the regions in only one curve than those in only two curves, and an area for the region in only the three curves that is typically similar to those for the regions in only one curve.

### 4.3. Comparison with Circles and venneuler

Using a variant of euler*APE*, our evaluation in Section 4.2 indicates that it is highly unlikely that there is 3-set data for which a good diagram can be drawn with circles. To verify this finding, we used the latest circle-based method venneuler version 1.1-0 to generate diagrams with circles for the 10,000 data items in L2. This method is the first to take a statistical approach and differs from euler*APE* in various ways. For instance, venneuler uses a numerical approximation method to compute the region areas and a steepest descent method with an approximate gradient to minimize its loss function *stress*. The accuracy of venneuler's diagrams was then compared with that of the diagrams generated by euler*APE* with circles and ellipses in Section 4.2.

For euler*APE*, a good diagram is a 3-Venn diagram with *diagError* ≤10^−6^ (Equation (3)). For venneuler, a good diagram is one with *stress* ≤10^−6^. Thus, to compare the accuracy of the diagrams generated by euler*APE* and venneuler, we computed: *stress* for the diagrams generated by euler*APE* using venneuler's version 1.1-0 source code, but euler*APE*'s analytic method to compute the region areas; *diagError* for the diagrams generated by venneuler using euler*APE*'s source code, but venneuler's numerical approximation to compute the region areas.

None of the diagrams generated by venneuler for the 10,000 data items in L2 had *stress* ≤10^−6^ or *diagError* ≤10^−6^. Thus, none of the diagrams were good according to venneuler's and euler*APE*'s diagram error measures. Also, only 64.5% (i.e., 6453/10,000) of the generated diagrams depicted all of the required regions. The other 35.5% (i.e., 3547/10,000) had one or more of the required regions missing.

**Figure 11:**
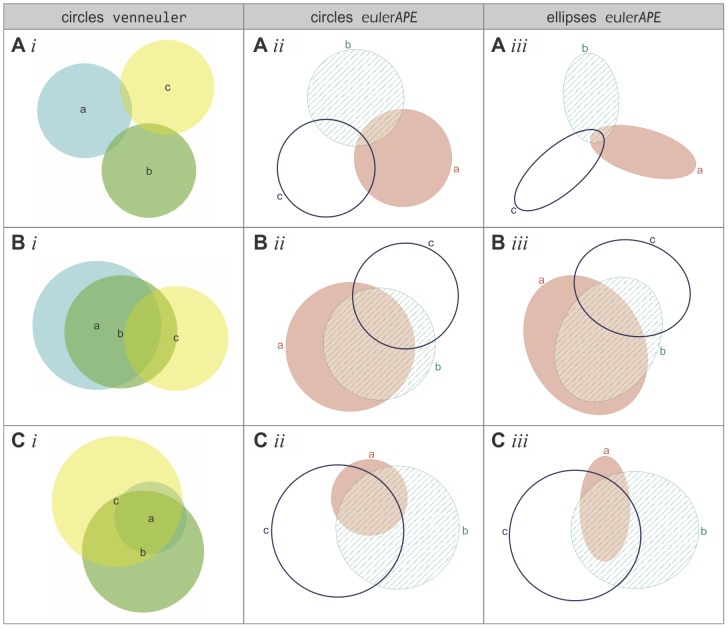
Examples of diagrams generated by venneuler and euler*APE* (circles and ellipses) for data in L2. Examples of diagrams generated with (*i*) circles by venneuler, (*ii*) circles by euler*APE*, and (*iii*) ellipses by euler*APE* for random 3-set data in L2. (A) Diagrams generated for data {*a* = 3491, *b* = 3409, *c* = 3503, *ab* = 120, *ac* = 114, *bc* = 132, *abc* = 126}. A*i* is missing region *abc* and has *stress*  = 5.69×10^−4^ and *diagError*  = 1.16×10^−2^. A*ii* and A*iii* have the required regions, one for every data set relation. A*ii* has *stress*  = 8.36×10^−3^ and *diagError*  = 2.63×10^−2^. A*iii* has *stress*  = 3.96×10^−12^ and *diagError*  = 6.55×10^−7^. (B) Diagrams generated for data {*a* = 45910, *b* = 3261, *c* = 45467, *ab* = 58845, *ac* = 3028, *bc* = 16406, *abc* = 18496}. B*i* is missing region *ac* and has *stress*  = 3.17×10^−3^ and *diagError*  = 2.07×10^−2^. There are two regions in B*i* depicting only *b*. B*ii* and B*iii* have the required regions, one for every data set relation. B*ii* has *stress*  = 2.13×10^−2^ and *diagError*  = 4.36×10^−2^. B*iii* has *stress*  = 3.43×10^−12^ and *diagError*  = 6.85×10^−7^. (C) Diagrams generated for data {*a* = 3664, *b* = 46743, *c* = 59811, *ab* = 1742, *ac* = 2099, *bc* = 17210, *abc* = 24504}. C*i*, C*ii* and C*iii* have the required regions, one for every data set relation. C*i* has *stress*  = 4.27×10^−3^ and *diagError*  = 2.30×10^−2^. C*ii* has *stress*  = 8.31×10^−3^ and *diagError*  = 2.44×10^−2^. C*iii* has *stress*  = 1.13×10^−12^ and *diagError*  = 4.03×10^−7^.

Figure 11A *i* and 10B*i* are examples of the diagrams generated by venneuler with missing regions. Both diagrams had a relatively low *stress* (5.69×10^−4^ and 3.17×10^−3^ respectively), close to that of a good diagram (i.e., *stress* ≤10^−6^). However, Figure 11A *i* was missing region *abc* (despite that its required area was larger than that of regions *ab* and *ac* and similar to that of region *bc*) and Figure 11B *i* was missing region *ac* (despite that its required area was similar to that of region *b*). Such diagrams are more misleading than ones with inaccurate region areas, as besides showing incorrect quantities, not all the required set relations are depicted. In contrast, the *diagError* for these diagrams was not so low (1.16×10^−2^ and 2.07×10^−2^ respectively). Some of venneuler's diagrams also had aesthetic features that could impede diagram comprehension [64]. For instance, Figure 11B *i* has two regions representing only *b*. These problems are not evident in euler*APE*'s diagrams *ii* and *iii* in Figure 11, as the diagram goodness measure and other checks during the optimization disallow the generation of such diagrams. When venneuler generated a diagram with all the required regions, the diagram was often misleading, as the region areas were inaccurate due to the limited degrees of freedom of circles. Figure 11C *i* is an example of such a diagram. According to the data for which this diagram was generated, region *a* had to be 2.1 times larger than region *ab* and 1.7 times larger than region *ac*. However, region *a* was smaller than both regions *ab* and *ac*. The *stress* of the diagram was low (*stress*  = 4.27×10^−3^, *diagError*  = 2.30×10^−2^), but greater than that of Figure 11A *i* and Figure 10B *i*, despite that the latter had missing regions and were thus more misleading.

The diagrams by euler*APE* with circles (*ii* in Figure 11) had inaccurate and misleading region areas like those of venneuler, but all depicted the required regions. All of euler*APE*'s diagrams with ellipses (*iii* in Figure 11) had the required regions as well as *stress* ≤10^−6^ and *diagError* ≤10^−6^ and were thus considered good by both venneuler's and euler*APE*'s error measures.

**Figure 12:**
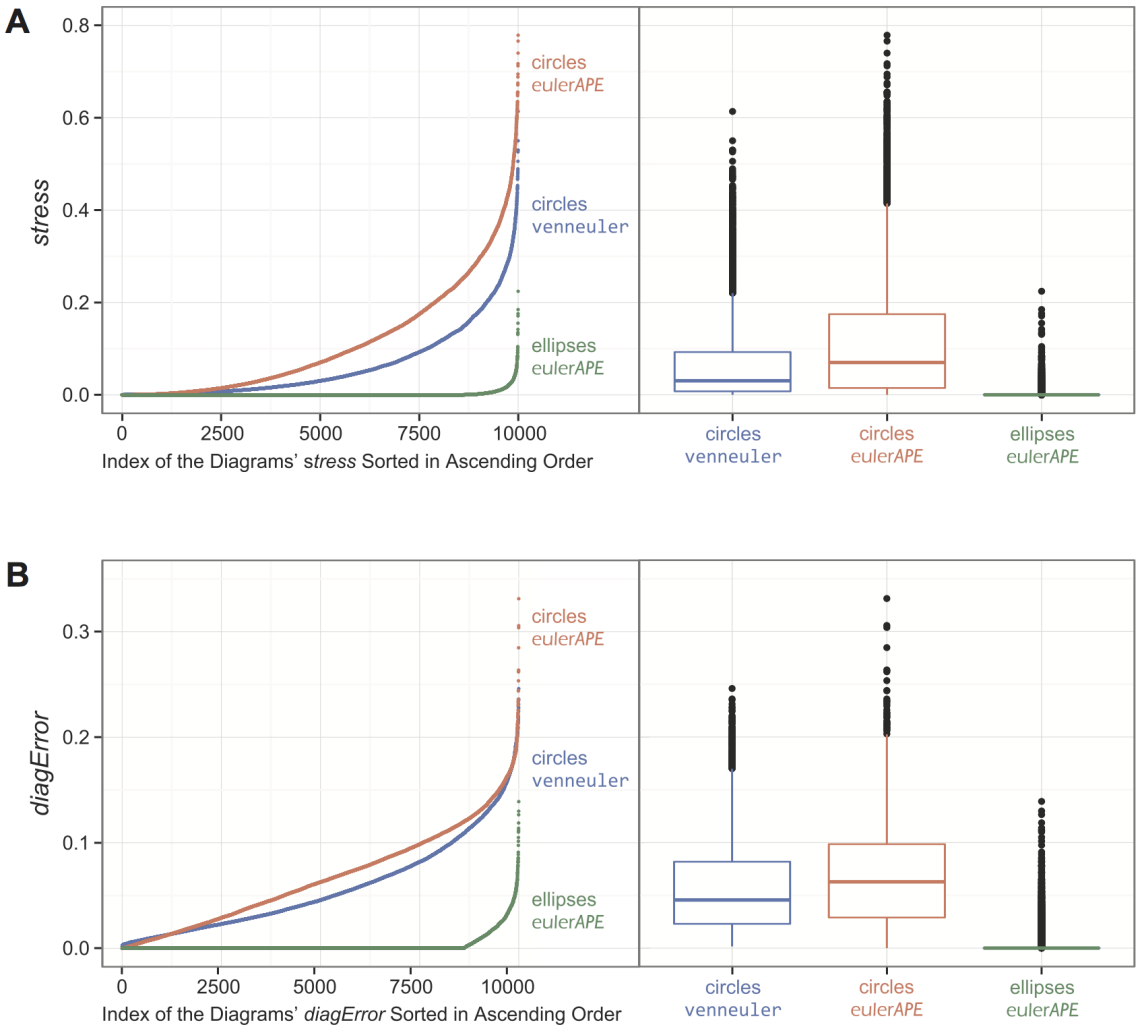
*Stress* and *diagError* of all the diagrams generated by venneuler and euler*APE* (circles and ellipses). The (A) *stress* and (B) *diagError* of all the diagrams generated with circles by venneuler, with circles by euler*APE* and with ellipses by euler*APE* for the 10,000 3-set data in L2. The 10,000 diagrams generated with circles by venneuler had *stress* in [3.77×10^−5^, 6.14×10^−1^] with median 3.04×10^−2^ and mean 6.41×10^−2^, and *diagError* in [1.56×10^−3^, 2.46×10^−1^] with median 4.56×10^−2^ and mean 5.73×10^−2^. The 10,000 diagrams generated with circles by euler*APE* had *stress* in [1.91×10^−10^, 7.79×10^−1^] with median 7.00×10^−2^ and mean 1.13×10^−1^, and *diagError* in [3.30×10^−6^, 3.31×10^−1^] with median 6.28×10^−2^ and mean 6.73×10^−2^. The 10,000 diagrams generated with ellipses by euler*APE* had *stress* in [3.98×10^−14^, 2.24×10^−1^] with median 7.59×10^−12^ and mean 1.17×10^−10^, and *diagError* in [6.00×10^−8^, 1.39×10^−1^] with median 8.00×10^−7^ and mean 2.94×10^−3^.

As shown in Figure 12A (for *stress*) and Figure 12B (for *diagError*), the majority of venneuler's diagrams had a lower *stress* and *diagError* than those of euler*APE*'s diagrams with circles (a lower *stress* for 8675/10,000 diagrams; a lower *diagError* for 6234/10,000 diagrams), but a greater *stress* and *diagError* than those of euler*APE*'s diagrams with ellipses (a greater *stress* for 9730/10,000 diagrams; a greater *diagError* for 9660/10,000 diagrams).

The differences between venneuler's diagrams and euler*APE*'s diagrams with ellipses were expected due to the limitations of circles in generating accurate diagrams for most data [30]. None of venneuler's diagrams were considered good by *stress* and *diagError*, but 8529 and 8607 of the 10,000 diagrams generated by euler*APE* with ellipses were considered good by respectively *stress* and *diagError* (the difference between the percentages of good diagrams by *stress* and *diagError* for euler*APE*'s diagrams with ellipse is not statistically significant—using R's pro.test with Yates' continuity correction disabled, ^χ2^(1) = 2.48, *p* = 0.12).

The differences between venneuler's and euler*APE*'s diagrams with circles could be less expected. A Friedman rank sum test for non-normal distributions and repeated-measure data revealed a significant effect of drawing method on *stress* (^χ2^(1) = 5402.3, *p*<2.2×10^−16^) and *diagError* (^χ2^(1) = 609.1, *p*<2.2×10^−16^). Post-hoc tests using Wilcoxon tests with Bonferroni correction showed significant differences between venneuler and euler*APE* with a large effect size on *stress* (*W* = 1763624, *Z* = −80.50, *p*<2.2×10^−16^, *r* = 0.57) and a medium effect size on *diagError* (*W* = 14730686, *Z* = −35.58, *p<*2.2×10^−16^, *r* = 0.25). So according to these measures venneuler's diagrams were more accurate than those of euler*APE*. However, while all of euler*APE*'s diagrams depicted the required regions, 35.5% of venneuler's diagrams had missing regions and yet 83.5% of these diagrams had a low stress (*stress*<10^−2^). So, euler*APE*'s diagrams could still be more helpful than those of venneuler as all the required set relations are depicted. Also, out of the 10,000 diagrams drawn by euler*APE* with circles, zero (i.e., 0%) had *diagError* ≤10^−6^ (Section 4.3), but 28 (i.e., 0.3%) had *stress* ≤10^−6^ (the difference between these percentages is statistically significant—using R's pro.test with Yates' continuity correction disabled, ^χ2^(1) = 28.04, *p* = 1.19×10^−7^). Thus, with 99% confidence, these *stress* results indicate that a good diagram with *stress* ≤10^−6^ can be generated with circles for 0.2% to 0.5% of random 3-set data by euler*APE* and for 0.0% to 0.1% of the same type of data by venneuler. The *diagError* of these diagrams that were considered good by *stress* was still relatively low and close to *diagError* ≤10^−6^.

This evaluation also revealed that if the required areas for the regions in only one curve are around twice as large as those for the regions in only two curves, and the area for the region in exactly the three curves is larger or as large as the areas for the regions in only one curve, then it is highly likely that a close to accurate area-proportional 3-Venn diagram drawn with circles exists.

With respect to the time taken to generate each diagram, venneuler was faster than euler*APE*. The median and mean generation time of venneuler were 0.6 seconds each, with a minimum of 0.4 seconds and a maximum of 1.0 second. The median and mean times for euler*APE* to generate a diagram with ellipses were 0.4 seconds and 5.3 seconds and with circles 3.2 seconds and 3.4 seconds. This could be due to the various differences between venneuler and euler*APE* (e.g., the method to compute the region areas; the way the optimization is run and terminated after a maximum of 200 iterations). Despite this, euler*APE* generates more accurate diagrams than venneuler and within a time that ensures users' attention is maintained (Section 4.2).

### 4.4. Comparison with Circles and Polygons, and Various Drawing Methods

Area-proportional 3-Venn diagrams are used extensively in various disciplines to facilitate data analysis, but often the diagrams are more misleading than helpful due to the limitations of the curve shapes used by current drawing methods. We investigated this further using real world medical data obtained from a BMC Medicine journal article [37]. Diagrams for this data were generated using most of the current drawing methods (Section ‘*Current Automatic Drawing Methods and Software*’). These were then analysed and compared with the diagram generated by euler*APE* using ellipses.

The selected article discusses the results from a web-based survey that assessed whether US trainees in family and internal medicine are aware of the complications, screening methods and therapy for chronic kidney disease (CKD). This survey data was comprised of sets *A*, *B* and *C*: trainees who claimed that secondary hyperparathyroidism is a complication of CKD (set *A*); trainees who screened by stage 3 of CKD (set *B*); trainees who commenced therapy or referred the patient to a specialist when parathyroid hormone (PTH) reached a level of PTH>70 ng/ml (set *C*). The set relations and associated quantitative data can be summarized as *ω* = {*A* = 0.25, *B* = 0.01, *C* = 0.11, *AB* = 0.10, *AC* = 0.29, *BC* = 0.03, *ABC* = 0.15}. To raise awareness that current trainees need further skills and guidelines to timely identify and manage patients with CKD, an area-proportional Venn diagram with respect to *ω* (Figure 3-D) was included in the article.

Diagrams with respect to *ω* were generated using eight circle-based drawing methods (from those listed and cited in Section 2.1), namely: **C1**, Stata's PVENN; **C2**, Venn Diagram Plotter; **C3**, 3 Circle Venn; **C4**, a module in PatternLab for proteomics; **C5**, BioVenn; **C6**, Vennerable circles; **C7**, venneuler; **C8**, Google Venn Charts. Other diagrams with respect to *ω* were generated using six polygon-based drawing methods (from those listed and cited in Section 2.2), namely: **P1**, VennMaster, with regular, circle-like polygons; **P2**, Vennerable triangles, with triangles; **P3**, DrawVenn, with rectilinear polygons; **P4**, Vennerable squares, with rectangular polygons; **P5**, Convex Venn-3, with 4-sided and 5-sided convex polygons; **P6**, DrawEuler, with irregular, non-convex polygons. All the diagrams are available in Figure 3, together with the diagram generated by **E**, euler*APE* with ellipses. The design of each diagram (e.g., labels, legend, colours, outlines, background) is precisely the same as that generated by the drawing method. Curve labels were only added to C2, P3 and P6 as no labels or legend are provided with the diagram. The numeric labels in euler*APE*'s diagram were added manually to illustrate how the diagram in the article would have looked like if it was drawn with ellipses.

The *diagError* was devised to compute the error of only those diagrams that depict all the required set relations, as diagrams with missing regions are more misleading than those with inaccurate region areas and should not be accepted altogether. Thus, Figure 3 shows the *diagError* of only those diagrams with seven regions interior to their curves as required by the data. For the other diagrams, the missing regions are noted. To calculate *diagError* using Equation (2), the region areas of the diagrams were computed using euler*APE*'s analytic method for those drawn with circles and standard geometry formulae for those drawn with polygons.

In Figure 3, we note that all the diagrams drawn with circles including D (the diagram in the article) have inaccurate region areas and are misleading. For instance, region *B* (1% in *ω*) is much larger than region *BC* (3% in *ω*), region *C* (11% in *ω*) is larger than regions *AB* (10% in *ω*) and *ABC* (15% in *ω*), and region *A* (25% in *ω*) in most diagrams is larger than region *AC* (29% in *ω*). Similar problems are also evident in C7, the diagram generated by the latest method venneuler. C3, generated by the first circle-based drawing method 3 Circle Venn, also has region *ABC* (15% in *ω*) similar in area to that of region *AC* (29% in *ω*). The same is evident in D, as D was generated by the method of C3. However, C8 is the most misleading and inaccurate, as region *BC* is missing and regions *B* and *AB* (respectively 1% and 10% in *ω*) are much larger than regions *C*, *AC* and *ABC* (respectively 11%, 29% and 15% in *ω*). With respect to *diagError*, the most accurate are C4 and C7 (*diagError*  = 0.03), followed by C2, C5, C6 (*diagError*  = 0.04) and C1 (*diagError*  = 0.05), and finally C3 and thus D (*diagError*  = 0.14). Due to the regularity and good continuation of circles, the curves are often easily distinguishable and identifiable. In a few cases (e.g., C3 and D), it is difficult to comprehend in which curves the regions are located. However, this is often down to design as, for instance, different unrelated colours are used for regions located in the same curve (e.g., C2, C3, C6).

In contrast, most of the diagrams with polygons are either accurate with *diagError* ≤10^−6^, as P3, P5, P6, or have region areas that are less misleading than those of diagrams with circles, as P2, P4. The latter is true as for instance, consistent with *ω*, region *B* is always the smallest and region *AC* is always the largest. The only diagram with missing regions (regions *C* and *BC*) is P1 generated by the non-deterministic method VennMaster. Since the curves are depicted as regular, circle-like polygons, VennMaster has the same limitations as others that use circles. Though the diagrams with polygons are more accurate than those with circles, the curves are non-smooth. So the curves are not easily identified [65] and are less likely to pop out as discrete and complete objects [2]. Curve identification is particularly difficult when, for instance: the curves met at bending points, as P5 and P6; the curves are partially concurrent, as P2, P3, P4; the curves are non-convex, as P6. Such features impede diagram comprehension [32], [64], making these diagrams accurate but not usable. Thus, the preference for the less accurate diagrams with circles instead of polygons.

Using ellipses, diagram E has region areas that are accurately and directly proportional to the quantities in *ω* (*diagError* ≤10^−6^). It is also easy to comprehend, as the curves are regular and have good continuation like circles. So ellipses can be more effective than both circles and polygons. This was also demonstrated with other real world data in Section ‘*Introduction*’, where Figure 2 illustrates the accurate and easy to comprehend diagrams generated by euler*APE* with ellipses as alternatives to the respective misleading diagrams drawn with circles in Figure 1A–C and the incomprehensible diagrams drawn with polygons in Figure 1D–F. Being the only method that uses ellipses, the effectiveness of ellipses could be the primary reason why euler*APE* is being used in various areas and why its diagrams are appearing in various journal articles (discussed in Section ‘*Introduction*’). The design of the diagrams adopted by euler*APE* is also different from that of other drawing methods, as euler*APE* uses a heterogeneous channel-based approach [2] whereby different feature types (i.e., outline, colour, texture) that are perceptually processed in parallel are used. In this way, none of the curve designs fuse perceptually at overlaps, and the curves and the regions are easily identified.

**Figure 13:**
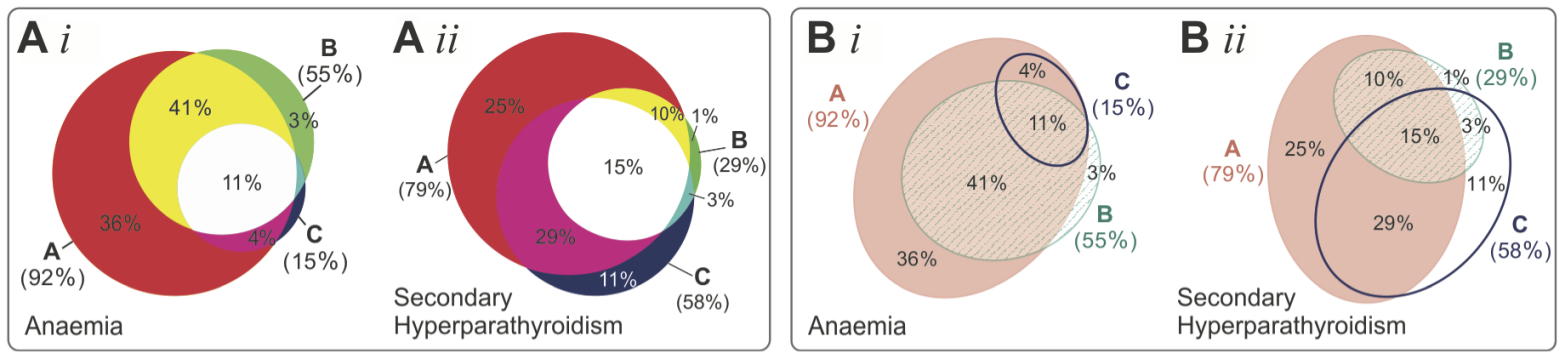
The figure in a medical journal article and the figure recreated with euler*APE*. (A) The figure with two Venn diagrams drawn with circles in a medical journal article [37]. This is a redrawing of Figure 5 in [37], previously published under a CC BY license. (B) The figure as it would have looked like if the diagrams were drawn with ellipses using euler*APE*. Labels for euler*APE*'s diagrams were added manually.

Another area-proportional 3-Venn diagram for the same data sets but for the management of anaemia rather than secondary hyperparathyroidism (so set *C* was based on the haemoglobin level rather than parathyroid hormone) was included in the article, as shown in Figure 13. As explained earlier, Figure 13A *ii* (replica of Figure 3-D) is misleading due to inaccuracies in the region areas. Figure 13A *i* could be more misleading as besides inaccuracies in the region areas (e.g., region *B* with 3% is larger than region *AC* with 4%; region *A* with 36% is larger than region *AB* with 41%), seven regions are shown when according to the data (i.e., {*A* = 0.36, *B* = 0.03, *C* = 0.00, *AB* = 0.41, *AC* = 0.04, *BC* = 0.00, *ABC* = 0.11}), regions *C* and *BC* should not be depicted. Currently euler*APE* draws highly accurate 3-Venn diagrams even when regions are very small and barely visible. So, Figure 13B *i*, generated by euler*APE* with respect to {*A* = 0.36, *B* = 0.03, *C* = 0.00001, *AB* = 0.41, *AC* = 0.04, *BC* = 0.00001, *ABC* = 0.11}, could be used instead of Figure 13A *i*. Looking at Figure 13B *i* and *ii*, we can easily note that though most participants claimed that anaemia and secondary hyperparathyroidism were complications of CKD (set *A*), timely screening (set *B*) was more common with (*i*) anaemia than (*ii*) secondary hyperparathyroidism, while commencement of therapy or referral to a specialist (set *C*) was more often delayed when diagnosis was based on the (*i*) haemoglobin level than on the (*ii*) parathyroid hormone level. Thus, Figure 13B could have been more effective than Figure 13A in raising awareness of the need for trainees to be provided with further guidelines in managing CKD.

## Conclusions

We have described euler*APE*, the first automated method for drawing area-proportional 3-Venn diagrams using ellipses. Previous methods used either circles or polygons. Circles are smooth and generate easy to comprehend diagrams, but are limited as they cannot draw accurate diagrams for most 3-set data. Polygons are flexible and generate accurate diagrams, but their non-smooth curves produce difficult to comprehend diagrams.

Our evaluation indicates that using ellipses and euler*APE*, accurate area-proportional 3-Venn diagrams can be drawn for a large majority of random 3-set data (86%, *N* = 10000), far more than is possible with the circles that are highly preferred over polygons. So curves that are smooth like circles but more general like ellipses should be considered whenever a diagram cannot be drawn accurately with circles. This finding opens a wider research question as to whether curves with different degrees of freedom such as circles, ellipses, ovals, *n*-ellipses, regular *n*-gons and irregular *n*-gons could be considered progressively from the most specific to the more general until a curve type that generates an accurate diagram for the required region areas is found [30].

The results of our evaluation also indicate great potential for using ellipses to draw area-proportional diagrams with more curves. However, first, further evaluation should be conducted to assess the effectiveness of ellipses and a method like euler*APE* in handling 3-set data that requires an area of zero for various regions in the diagram. Following this, characteristics of different types of quantitative 3-set data that can or cannot be depicted accurately with an area-proportional diagram drawn with ellipses should be determined, and analytic methods that identify whether a diagram can be drawn accurately for the given data should be formalized.

Apart from the shape of the curves, diagram design features (e.g., colours, labelling strategies) can also facilitate or impede understanding of the diagram and the depicted data. The effect of such features and the possible benefits of adding interaction should be investigated. Other features that could aid understanding for users with different abilities (e.g., spatial and numeracy abilities) should also be identified.

A number of the studies could be conducted to understand: how such diagrams are processed perceptually and cognitively; how region areas are perceived; the effect of the shape of the regions and curves on area judgement; what discrepancies in areas are not noticeable; whether perceptual scaling measures like those proposed for map symbols in cartography [66], but highly criticized by Tufte [67], aid or hinder area judgement in these diagrams. The findings of these studies will aid in the identification of diagrams whose region area errors are not human detectable. In this way, an inaccurate diagram drawn with ellipses for the required data could be considered accurate for human use and perception and so, it could be drawn with ellipses rather than other more complex curves with less desirable features, such as irregular and jagged polygons. A study should also determine whether numeric labels in the regions could conceal errors in region areas, thus allowing the use of smooth curves.

Following these studies, aesthetic criteria, metrics and cognitive measures as well as perceptual and design guidelines defining an effective, good diagram for human use that facilitates comprehension and reasoning should be formalized and prioritized. A variant of euler*APE* should then be devised to optimize such measures, such that a diagram that is the best compromise between region area accuracy and aesthetics is generated. Such a diagram should ideally have all the important aesthetic features and none of its region area inaccuracies should be noticeable to the human user. This would be particularly important for data for which an accurate diagram with specific aesthetic features cannot be drawn. With such a compromise, an inaccurate diagram with smooth curves whose errors are not human detectable could be generated.

It might also be interesting to assess the effectiveness of allowing users to select aspects of the diagram that they consider important and they would like to optimize. Such aspects could include aesthetic features, such as the shape of certain curves or the accuracy of the regions.
